# *In silico* framework for designing and validating a multi-stage subunit vaccine against Tuberculosis using reverse vaccinology approach

**DOI:** 10.1371/journal.pone.0356788

**Published:** 2026-08-27

**Authors:** Ayesha Liaqat, Kubra Dastgir, Muhammad Sajjad, Hafiz Muzzammel Rehman, Muhammad Waheed Akhtar

**Affiliations:** 1 School of Biological Sciences, University of the Punjab, Lahore, Pakistan; 2 School of Biochemistry and Biotechnology, University of the Punjab, Lahore, Pakistan; NHS Blood and Transplant, UNITED KINGDOM OF GREAT BRITAIN AND NORTHERN IRELAND

## Abstract

Tuberculosis (TB), caused by *Mycobacterium tuberculosis (Mtb)*, remains a critical public health concern due to the limited efficacy of the Bacillus Calmette-Guerin (BCG) vaccine, the only WHO-approved vaccine so far. Based on the reverse vaccinology approach, this study involves *in silico* prediction of a fusion constructed from four highly immunogenic antigens from the latent, early, and active stages of the disease. The fusion named TetraFuVac11 consists of the complete sequences of the antigens CFP-7 and EspC and the truncated sequences of the antigens HspX and Hrp1. Major Histocompatibility Complex II (MHC II) binding Th-cell-specific epitopes were predicted through tools provided in the Immune Epitope Database (IEDB). The designed fusion molecule was found to be antigenic, non-allergenic and non-toxic. The instability index II and the GRAND Average of Hydropathy values were predicted to be 29.51 and −0.182, respectively. The refinement of the predicted 3D structure resulted in an improved stereochemical profile. The Z-score was predicted to be −6.8, and the ERRAT score was improved from 94.928 for the unrefined model to 97.1591 for the refined model. The data obtained from molecular dynamics (MD) simulations and Normal Mode Analysis (NMA) of the docked complex between the refined fusion protein and Toll-like Receptor 4 (TLR4) demonstrated a s interaction. The fusion construct was successfully predicted to be cloned into the pET-28a(+) vector to make a recombinant plasmid. The predicted solubility of the fusion protein exceeded the threshold values, indicating soluble expression in *Escherichia coli.* Finally, based on the encouraging *in silico* data, the proposed construct could serve as a potential vaccine candidate for detailed experimental validation towards developing an *Mtb*-specific vaccine.

## Introduction

Tuberculosis, caused by *Mycobacterium tuberculosis (Mtb),* is a highly contagious communicable disease that has been causing morbidity and mortality for many years. In the year 2024, according to a WHO report (2025), TB caused the deaths of 1.23 million people around the globe. This number included 15.9%, 34.4%, and 49.7% of children, women, and men, respectively. In 2024, around 10.7 million individuals contracted TB infection, which included 5.8% of HIV patients [1]. About 25% of individuals worldwide have been infected with *Mtb*, and they may progress to active infection at any stage of life [2]. Before the emergence of COVID-19, TB accounted for the highest mortality rate by any single infectious microorganism for many years [3]. Over the past few years, the incidence of cases involving multidrug-resistant TB (MDR-TB) has skyrocketed, making it the leading cause of death due to antimicrobial drug resistance [4]. BCG, the only WHO-approved preventive vaccine for TB disease, fails to provide sufficient immunological protection after 10 years of administration [5]. Moreover, the immunological response provided by the BCG vaccine is highly variable, ranging from 80% protection to no protection at all against pulmonary TB [6]. A highly specific subunit vaccine can prove to be an effective strategy to minimize the global disease burden of TB [7].

The absence of latency-associated antigens in the traditional BCG vaccine highlights a critical gap in current clinical research against pulmonary tuberculosis. Our designed fusion construct directly addresses this challenge by incorporating stage-specific antigens. HspX (Rv2031c) is expressed during the latency phase of *Mtb* infection. It has been identified as a key contributor to antibody and cellular immune responses, making it a promising target for *Mtb* vaccine [8]. It induces the activation of multiple Th1-associated cytokines, including TNF-alpha and IFN-gamma, upon recognition by CD4 + T cells [9]. HspX has also been reported to induce the release of IFN-gamma from PBMCs stimulated with *Mtb* antigens [10]. Rv2626c, also known as hypoxic response protein, or Hrp1, is expressed during the latent phase of *Mtb* infection [11]. This protein enables differentiation of the latent from the active TB patients because it is exclusively expressed during the latent phase of infection [12]. Rv2626c has also been reported to upregulate the secretion of TGF-β, IFN-γ, IL-10, IL-4, and IL-2 [13]. ESAT-6-like protein, also known as TB10.4 or CFP7 (Rv0288), is a low molecular weight protein expressed during the early phase of *Mtb* infection. This immunodominant protein is heavily embedded with numerous T-cell epitopes, making it a suitable candidate for vaccine design [14]. Another protein, EspC (Rv3615c), is one of the highly immunogenic proteins of *Mtb,* secreted through the ESX-1 secretion system during active TB infection [15]. The Immunological potential of EspC and EspB was demonstrated by their ability to elevate IL-4, IFN-γ, and IgG production in a Balb/C mice model [16]. TriFu64, a subunit vaccine generated by fusing Rv2628, EsxN, and PPE42 antigens, has been reported to reduce bacterial load in *Mtb*-infected mouse models [17].

Recently, computational vaccine design using the reverse vaccinology approach has gained importance in looking for novel vaccine candidates for tuberculosis. Previously, epitopes from Rv3804c, Rv2608, Rv0125, and Rv2684 *Mtb* antigens were integrated into a single subunit vaccine candidate, and *in silico* immune simulation analysis predicted increased expression of immunoglobulins, helper T cells, and cytotoxic T cells [18]. Another study formulated a multi-epitope subunit vaccine construct utilizing EsxA, EspA, LppX, LprA, PPE18, Mpt63, EsxB, and EspC antigens and predicted its overall stability, safety, antigenicity, and immunogenicity profiles through a rigorous *in silico* analysis [19]. By employing immunoinformatics and a subtractive proteomic strategy, a designed multi-epitope vaccine construct showed enhanced population coverage, improved antigenicity, and an appreciable binding affinity with TLR-4, MHC I, and MHC II [20]. A multi-epitope vaccine construct produced by coupling epitopes from PPE68, EspC, PE_PGRS17, RpfD, RpfC, and LDT4 was predicted to have a stable structure and the potential to elicit immune responses [21]. In another study, a fusion construct consisting of 34 epitopes from B cells, cytotoxic T lymphocytes, and helper T lymphocytes of PP13138R was predicted to elicit a cell-mediated immune response upon recognition by TLR4. This construct enhanced the production of IFN-ɣ in PBMCs from healthy, active, and latent TB donors [22].

Several subunit vaccine candidates are currently undergoing clinical evaluation due to their ability to elicit an effective immune response. M72/AS01, a subunit vaccine comprising a fusion of MTB32A and MTB39A antigens, was intended to serve as a booster for BCG to enhance immunological protection in adults and adolescents [23]. Clinical phase 2b trials showed M72/AS01 reduced the risk of latent TB progressing to the active state by 54.0%, with efficacy persisting for three years [24,25]. In another phase 2 trial among HIV patients, the vaccine was found to be safe and immunogenic, which ultimately led to its registration for phase 3 clinical trials [26]. Similarly, H56: IC31 vaccine containing Rv2660c, ESAT-6, and Ag85b antigens, was designed as a booster to BCG to strengthen its effect [27]. Although the vaccine was found to be safe and immunogenic, it showed limited potential for preventing disease relapse when administered at the end of TB treatment in phase 2b trials [28]. Even though M72/AS01 and H56:IC31 have demonstrated encouraging efficacy in clinical trials, a substantial proportion of individuals with latent TB infection (LTBI) still revert to the active state, underscoring the need for a multistage antigenic combination. The current study aims to design a single fusion molecule incorporating the multistage antigens HspX, CFP7, Hrp1, and EspC, utilizing a reverse vaccinology approach to assess its potential to serve as a subunit vaccine candidate against tuberculosis.

## Materials and methods

### Retrieval of protein sequences of the selected antigens

*Mycobacterium tuberculosis* H37Rv, belonging to phylogenetic lineage 4, is a well-characterized strain harbouring a plethora of immunogenic antigens [29]. Uniprot (https://www.uniprot.org/) is a comprehensive database for obtaining sequence information about various proteins [30]. The sequences of HspX (accession no: P9WMK1), CFP7 (accession no: P9WNK3), Hrp1 (accession no: P9WJA3), and EspC (accession no: P9WJD7) antigens were retrieved in FASTA format. For the construction of a fusion molecule, complete-length proteins of CFP7 and EspC and epitope-rich truncated forms of HspX and Hrp1 (tnHspX and tnHrp1) were used. HspX (16 kDa) was truncated by removing 20 residues from the N-terminal and 28 residues from the C-terminal, and it was named tnHspX (10.5 kDa). Similarly, Hrp1 (15.5 kDa) was truncated by removing 20 residues from the N-terminal and 3 from the C-terminal, and it was named tnHrp1 (13.5 kDa). These four proteins were joined together through -G-S- linkers. The addition of these flexible linkers may prevent the formation of rigid secondary structures (α-helices and β-sheets) at the junctions of antigens. They may also minimize steric clashes, enabling each antigen to fold independently into its biologically active conformation [31]. The hydrophilic nature of serine could also contribute to the solubility of the recombinant product [32]. The fusion protein was named TetraFuVac11. The final amino acid sequence of the TetraFuVac11 fusion construct is provided in the S1 File.

### Toxicity, antigenicity, and allergenicity of the Th1-cell-specific epitopes

The sequences of Th1-cell-specific epitopes of HspX, CFP7 and Hrp1 were obtained from the Immune Epitope Database (IEDB), a valuable resource to explore T and B cell epitopes of an antigen, as reported previously [33–35]. For EspC and tnHrp1, binding affinity for MHC II was predicted using various tools given on IEDB Analysis Resources (IEDB-AR) (https://tools.iedb.org/main/). The allelic variants chosen for HLA II binding affinity include an array of 6 HLA-DP, 15 HLA-DR, and 6 HLA-DQ alleles. This panel also includes supertypes that are present in the majority of the population with different ethnic origins [36]. Firstly, the TepiTool software was used for predicting MHC II binding epitopes [31]. The predicted epitopes with a < 1% percentile rank underwent further *in silico* prediction by the NetMHCIIpan 4.0 predictor (https://services.healthtech.dtu.dk/services/NetMHCIIpan-4.0/), which is based on artificial neural networks [37]. Using the NetMHCIIpan 4.0 predictor, only those peptides were chosen that exhibited a minimum rank value of 1% and <1%. Antigenicity of the epitopes was evaluated with the VaxiJen v2.0 tool (http://www.ddg-pharmfac.net/vaxijen/VaxiJen/VaxiJen.html) against a 0.4 threshold [38]. It is a popular tool for predicting antigenicity of whole proteins or smaller peptides using an alignment-independent approach. Allergenicity of the epitopes was predicted with AllerTOP v2.1 (https://www.ddg-pharmfac.net/AllerTOP/method.html), which maps allergenic peptides and proteins based on physicochemical properties [39]. The toxicity of the epitopes was predicted using the ToxinPred server (https://webs.iiitd.edu.in/raghava/toxinpred/), which uses a Support Vector Machine (SVM) model to predict toxic peptides [40]. Lastly, the population coverage of the predicted epitopes was calculated using the Population Coverage tool (https://tools.iedb.org/population) on IEDB against the selected panel of HLA alleles [31]. After the prediction of Th1-cell-specific epitopes, tnHspX, CFP7, tnHrp1, and EspC were joined together through -G-S- linkers.

### Prediction of toxicity, antigenicity, and allergenicity of TetraFuVac11

A successful vaccine should be non-toxic, antigenic, and non-allergenic. Toxicity, antigenicity, and allergenicity of the TetraFuVac11 fusion protein were estimated using ToxinPred, VaxiJen v2.0, and AllerTOP v2.1, respectively. Additionally, the allergenicity of the fusion molecule was also evaluated using the AllergenFP v1.1 server (https://ddg-pharmfac.net/AllergenFP/) [41].

### Prediction of physicochemical attributes

The physicochemical properties of the TetraFuVac11 molecule were analyzed using the ProtParam server (https://web.expasy.org/protparam/). This tool calculates the chemical and physical properties of a protein on the basis of its primary sequence. The isoelectric point, instability index, and hydropathy score of the fusion protein were estimated using the ProtParam server [42].

### Prediction of secondary and tertiary structures of TetraFuVac11

For determining the two-dimensional structure of the TetraFuVac11 molecule, the PSIPRED tool (https://bio.tools/psipred) was used. This tool combines Artificial Neural Networks (ANNs) and phylogenetic analysis to generate the 2D structure of a protein [43]. The 2D structure of TetraFuVac11 was also generated with the PDBsum web server (https://www.ebi.ac.uk/thornton-srv/databases/pdbsum/). Using the PDB coordinate data, this tool also shows the distribution of α-helices, β-sheets, and loops in the protein molecule [44].

The three-dimensional structure of TetraFuVac11 was modelled with AlphaFold2 (https://alphafold.ebi.ac.uk/). It is a powerful structural biology tool for predicting a highly accurate 3D structure from a primary protein sequence using a machine learning method [45].

### Structure refinement and *in silico* prediction

The GalaxyRefine web server (https://galaxy.seoklab.org) was employed to refine the 3D model of TetraFuVac11, predicted by AlphaFold2. It is a universally adopted web server that improves the overall quality of a predicted protein structure by minimizing steric clashes and enhancing hydrogen bond interactions [46]. For subsequent analysis, a protein model with the highest Ramachandran scores and a favorable MolProbity score was selected.

The *in silico* prediction of the 3D structure was performed by tools available in SAVES v6.1 [47]. Using the Procheck (https://saves.mbi.ucla.edu/) web server, a Ramachandran plot was generated to investigate the folding pattern of the fusion protein. The fusion construct was also validated by the ERRAT score, which predicts model quality by its overall quality factor. It compares the non-bonded interactions in a predicted protein structure to experimentally determined values [48]. The fusion protein structure was also validated by energy plots generated by ProSA-web (https://prosa.services.came.sbg.ac.at/prosa.php). It is an extensively used online server to evaluate inaccuracies in the predicted 3D structures of proteins [49].

### Prediction of linear and discontinuous B-cell epitopes

Predicting B-cell epitopes is also a pivotal part of vaccine development because they directly participate in antibody production during the humoral immune response. For the prediction of linear B-cell epitopes, the ABCpred (http://webs.iiitd.edu.in/raghava/ABCpred/) tool was employed [50]. The epitopes showing an epitope score > 0.7 against a threshold of 0.5 were selected. The predicted epitopes were further screened for toxicity, antigenicity, and allergenicity using ToxinPred, VaxiJen v2.0, and AllerTOP v2.1, respectively.

With the ElliPro web server (https://tools.iedb.org/ellipro/result/predict/), the conformational B-cell epitopes of the fusion protein were predicted. This tool clusters neighbouring residues within a specified proximity and estimates the Protrusion Index (PI) for all residues in a protein for predicting conformational epitopes. It also ranks epitopes based on their average PI score [51].

### Disulfide engineering of TetraFuVac11

Disulfide bonds are crucial to the structural integrity of any designed vaccine construct. Using the default parameters of Disulfide by Design 2 v2.13 software (http://cptweb.cpt.wayne.edu/DbD2/), disulfide bonds were introduced in the 3D structure of the TetraFuVac11 molecule. It is a widely used software to scan residues capable of establishing disulfide bridges. The Cα-Cβ-Sγ angle was set to 114.6° ± 10 and the χ3 angle to −87° or +97° (± 30). Residue pairs with bond energy <2.2 kcal/mol were chosen for cysteine mutation [52,53].

### Molecular docking of TetraFuVac11 with TLR4

Molecular docking helps to study key interactions between the vaccine and receptor molecules. From the Protein Databank (https://www.rcsb.org/), the PDB structure of the Toll-like receptor 4 (TLR4) with the 4G8A PDB ID was downloaded. The water molecules and ligands were removed from TLR4 using PyMOL. TLR4 was then docked against the designed TetraFuVac11 molecule using the HDOCK server (http://hdock.phys.hust.edu.cn/) [54]. PRODIGY (PROtein binDIng enerGY prediction), an online tool by HADDOCK *(*https://rascar.science.uu.nl/prodigy/), was used to estimate the binding free energy (ΔG) and dissociation constant (Kd) of the docked complex [55]. It calculates the binding energy of a docked complex under static conditions, using the density of interfacial contacts and non-interacting surfaces. The initial binding energy of the static docked complex configuration was also estimated by the HawkDock server (http://cadd.zju.edu.cn/hawkdock/) [56]. Docking interactions were analyzed by the PDBsum web tool (https://www.ebi.ac.uk/thornton-srv/databases/pdbsum/). PDBsum serves as a cornerstone for interface analysis of docked protein complexes [44].

### Molecular dynamics simulations and Normal Mode Analysis

MD simulations were performed using the Schrödinger Desmond module [57] to analyze the structural stability and dynamic binding behaviour of the Fusion-TetraFuVac11 docked complex. The Simple Point-Charge (SPC) water model was employed for the solvation of the docked complex, while neutralization was done with Na^+^/Cl^-^ ions. Under the NPT ensemble, energy minimization of the docked complex was performed to avoid any high-energy structural constraints. MD simulations were carried out for 100 ns at 300 K temperature and 1 atmospheric pressure using the OPLS4 force field. RMSF, RMSD, and SSE plots were obtained to assess stability of the docked complex. The binding free energy of the TetraFuVac11-TLR4 docked complex was estimated using the Molecular Mechanics-Generalized Born Surface Area (MM-GBSA) approach.

Normal Mode Analysis (NMA) of the docked complex was performed using iMODS (https://imods.iqf.csic.es/)*.* It generates several distinct plots for the docked complex, including a deformity plot, eigenvalue, B-factor mobility plot, elastic network model, variance, and covariance plots. The flexible regions of the docked complex are shown in the deformity plot, and the amount of energy needed to deform a particular structure is shown in the form of an eigenvalue. The B-factor profile shows atomic fluctuations in comparison to the equilibrium conformation. The elastic network model helps study the flexibility in the protein model by representing it as an interconnected network of strings. The variance plot shows the contribution of each normal mode to the overall flexibility of the protein model. Lastly, the covariance plot shows the relationship between the motions of constituent amino acids in a protein model in a correlation matrix [58].

### Immune simulations

The probable antibody and cellular immune response spectrum against the TetraFuVac11 molecule was analyzed by the C-immSim tool (https://kraken.iac.rm.cnr.it/C-IMMSIM/index.php). It predicts the immune spectrum in mammals by applying agent-based modeling (ABM) and a Position-specific Scoring Matrix (PSSM) [59]. The immune profiling of the vaccine molecule was conducted by injecting three doses (a primary dose followed by two booster doses) over a four-week time interval. The time periods of 1, 84, and 168 were selected, where each interval corresponds to 8 hours of real life. The injection volume was maintained at 50 µl while the remaining parameters were set to their default values, including 12345 seed speed, no LPS, and 1000 simulation steps.

### Prediction of surface accessibility

The ProtScale tool (https://web.expasy.org/protscale/) by the ExPASy web server was used for predicting surface accessible regions of TetraFuVac11 [60]. To choose % accessible residues, the protein sequence was scanned using a 9-residue sliding window. Only windows with a 4.122–7.244 accessibility score and a mean of +1 standard deviation were selected. The sequence-based surface accessibility was also analyzed by NetSurfP-2.0 (https://services.healthtech.dtu.dk/services/NetSurfP-2.0/). It is a deep learning tool to evaluate relative surface accessibility (RSA) against a threshold of 25% [61].

### *In silico* cloning and solubility prediction

The open reading frames of HspX, CFP-7, EspC, and Hrp1 retrieved from MycoBrowser (https://mycobrowser.epfl.ch/) [62] were fused through the linkers. With Rare Codon Caltor (https://people.mbi.ucla.edu/sumchan/caltor.html), the codons were optimized for successful transformation in the *E. coli* host. The *Nde*I restriction enzyme was introduced to the N-terminus and *Bam*HI to the C-terminus of the fusion sequence. For *in silico* cloning, the optimized codon sequence of the TetraFuVac11 protein was cloned into the pET-28a(+) vector through SnapGene software (https://www.snapgene.com/).

Solubility of the fusion molecule when expressed in *E. coli* was predicted with SoluProt 1.0 (https://loschmidt.chemi.muni.cz/soluprot/) with a threshold value of 0.5 and Protein-Sol (https://protein-sol.manchester.ac.uk/) against a 0.45 threshold value [63,64].

## Results

### Retrieval of protein sequences of the selected antigens

The complete sequence of amino acid residues of HspX, CFP7, Hrp1, and EspC was obtained from UniProt with the following accession numbers: P9WMK1, P9WNK3, P9WJA3, and P9WJD7. Complete-length proteins of CFP7 (10.3 kDa) and EspC (10.7 kDa) were used for the construction of the TetraFuVac11 molecule. For HspX and Hrp1, truncations were made by removing non-epitopic segments from the N- and C-termini. Both of the truncated proteins showed improved surface accessibility of the epitopes in the 3D structure predicted by AlphaFold2 (S2 Fig). The use of tnHspX and tnHrp1 also helped to maintain the molecular size of the fusion construct within a good working range. All the antigens were then joined together through -G-S- linkers into a single fusion molecule named as TetraFuVac11.

### Toxicity, antigenicity, and allergenicity of Th1 cell-specific epitopes

**Fig 1:**
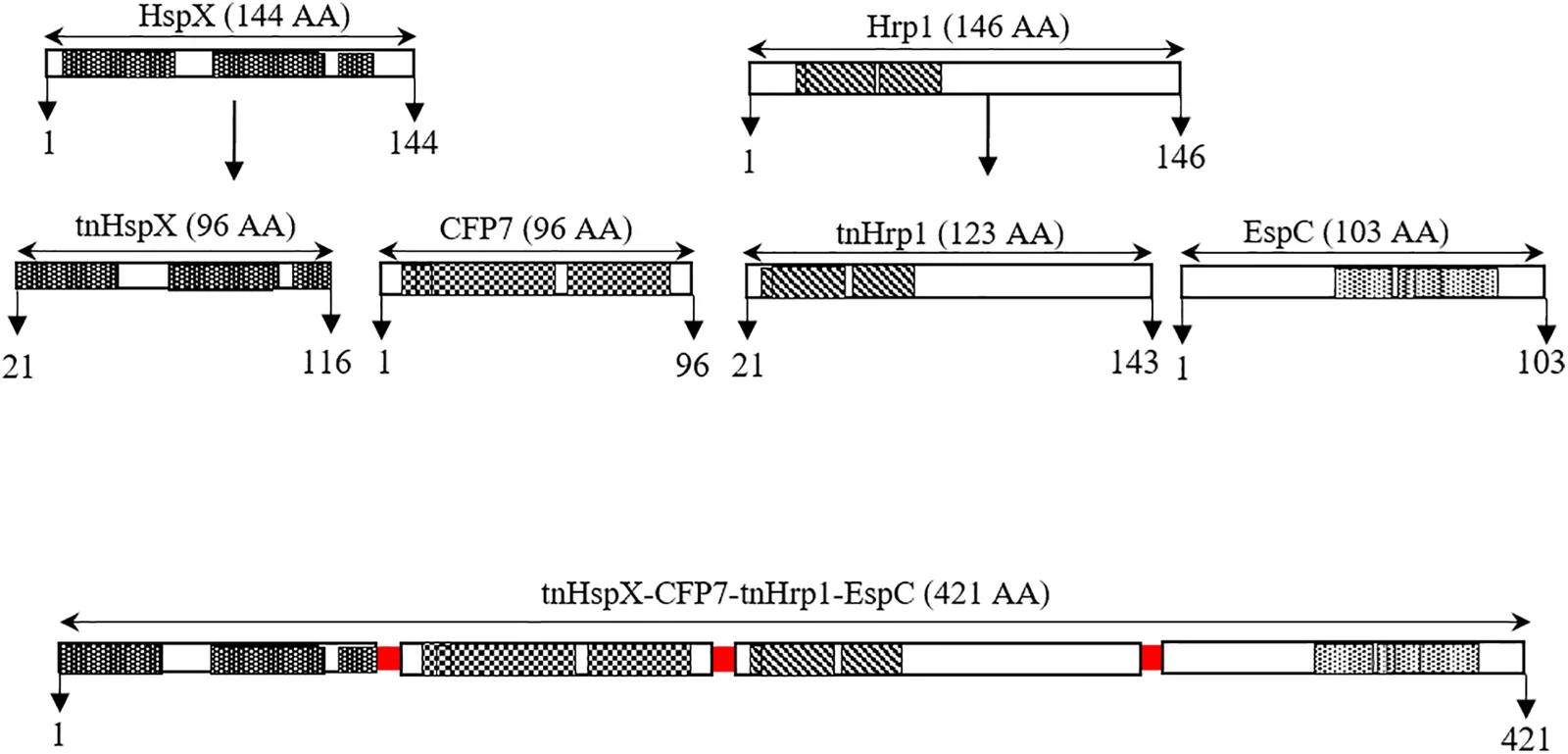
Construction of TetraFuVac11 fusion protein from CFP7, EspC, tnHspX, and tnHrp1. The location of epitopes in each antigen is highlighted; the red blocks represent the linkers.

**Fig 2:**
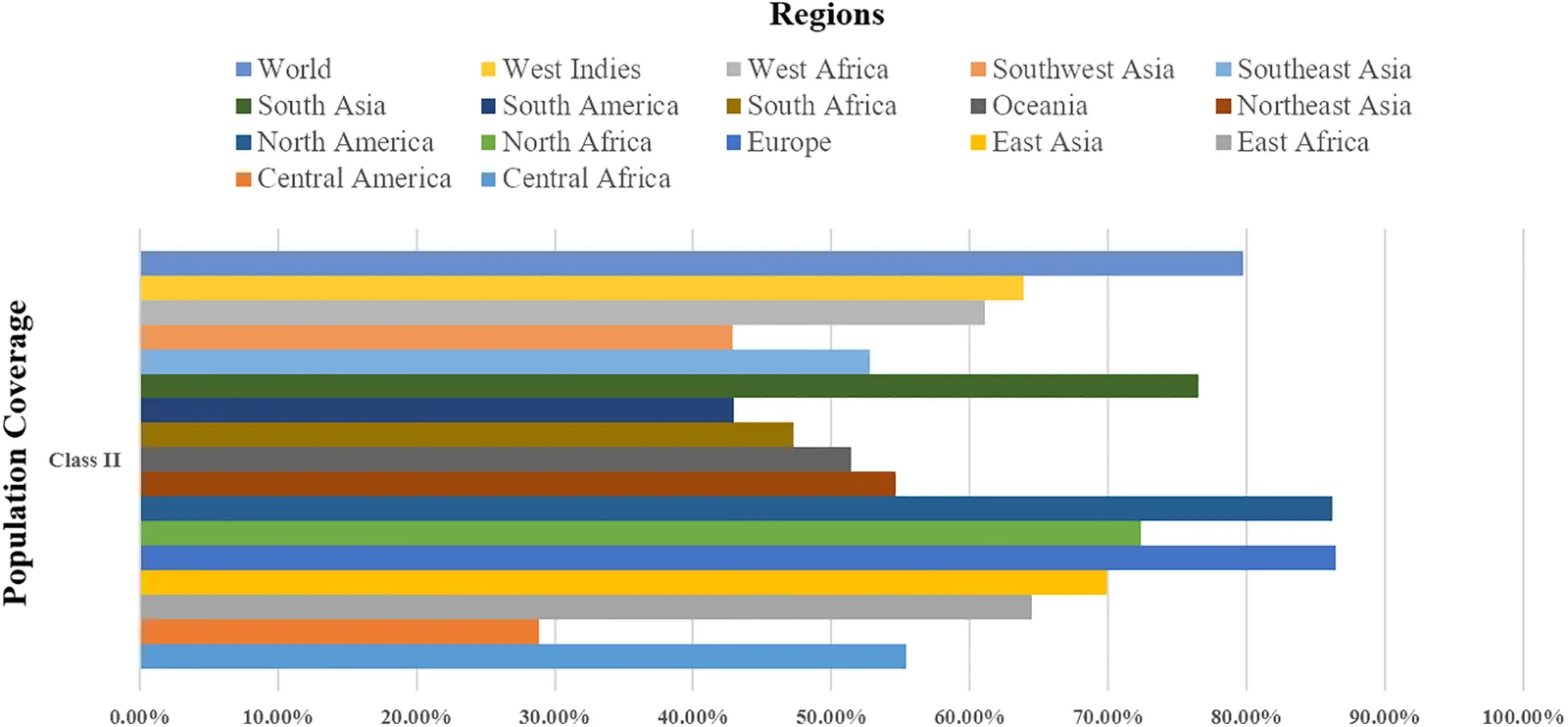
Population coverage analysis of the MHC II-binding epitopes of the TetraFuVac11 vaccine construct as predicted by the Population Coverage tool of IEDB.

According to IEDB analysis, 16, 14, and 3 T-cell-specific epitopes were reported for CFP7, HspX, and Hrp1, respectively (S3 Table). For EspC and tnHrp1, MHC II-binding T-cell-specific epitopes with a < 1% percentile rank, as determined by the Tepitool web server, were selected for further analysis (S4 Table). Further validation by the NetMHCIIpan 4.0 predictor yielded 8 Th1-cell-specific epitopes for EspC. For tnHrp1, 10 epitopes were predicted using the same tools. Among the reported CFP epitopes, 6 and 8 were predicted to be antigenic and non-allergenic, respectively, whereas all were found to be non-toxic. For HspX, 9 were antigenic, 6 were non-allergenic, and all were non-toxic. From the predicted epitopes of EspC, 6 and 7 were antigenic and non-allergenic, respectively, and all were non-toxic (S5 Table). Of the reported three Hrp1 epitopes, all were antigenic and non-toxic, and 2 were non-allergenic (S3 Table). However, out of the predicted epitopes of tnHrp1, 2 were antigenic, 6 were non-allergenic, and all were non-toxic (S5 Table). Overall, four for CFP7, five for HspX, and two for Hrp1 were predicted to be non-toxic, antigenic, and non-allergenic. From the predicted epitopes, five for EspC and one for tnHrp1 were found to be non-toxic, antigenic, and non-allergenic. Fig 1 shows the schematic location of antigenic, non-allergenic, and non-toxic Th-cell-specific epitopes in the component antigens and the TetraFuVac11 protein. Population coverage analysis for MHC II-binding epitopes of tnHspX, CFP7, EspC, and tnHrp1 in the fusion protein showed 79.75% coverage globally, while population coverage data for the other regions are shown in Fig 2.

### Prediction of toxicity, antigenicity, and allergenicity of TetraFuVac11

The fusion protein was predicted to be an antigen by VaxiJen v2.0, as it yielded a 0.594 score against a threshold of 0.4. The fusion protein was inferred as a probable non-allergen by AllerTOP v2.1 and AllergenFP v1.1. It was inferred to be non-toxic by the ToxinPred server. These results suggest that the fusion protein possesses a safer biosafety profile for *in vivo* administration.

### Prediction of physicochemical attributes

Physicochemical attributes of TetraFuVac11 were analyzed by the ExPASY ProtParam tool. The molecular weight of the fusion molecule was estimated as 45.5 kDa. A pI of 4.79 for the fusion protein shows its acidic nature. The stability of the fusion protein was depicted by a favourable instability index II of 29.51. The hydrophilic nature of the fusion molecule was indicated by a Grand Average of Hydropathy (GRAVY) value of −0.182.

### Prediction of secondary and tertiary structures of TetraFuVac11

**Fig 3:**
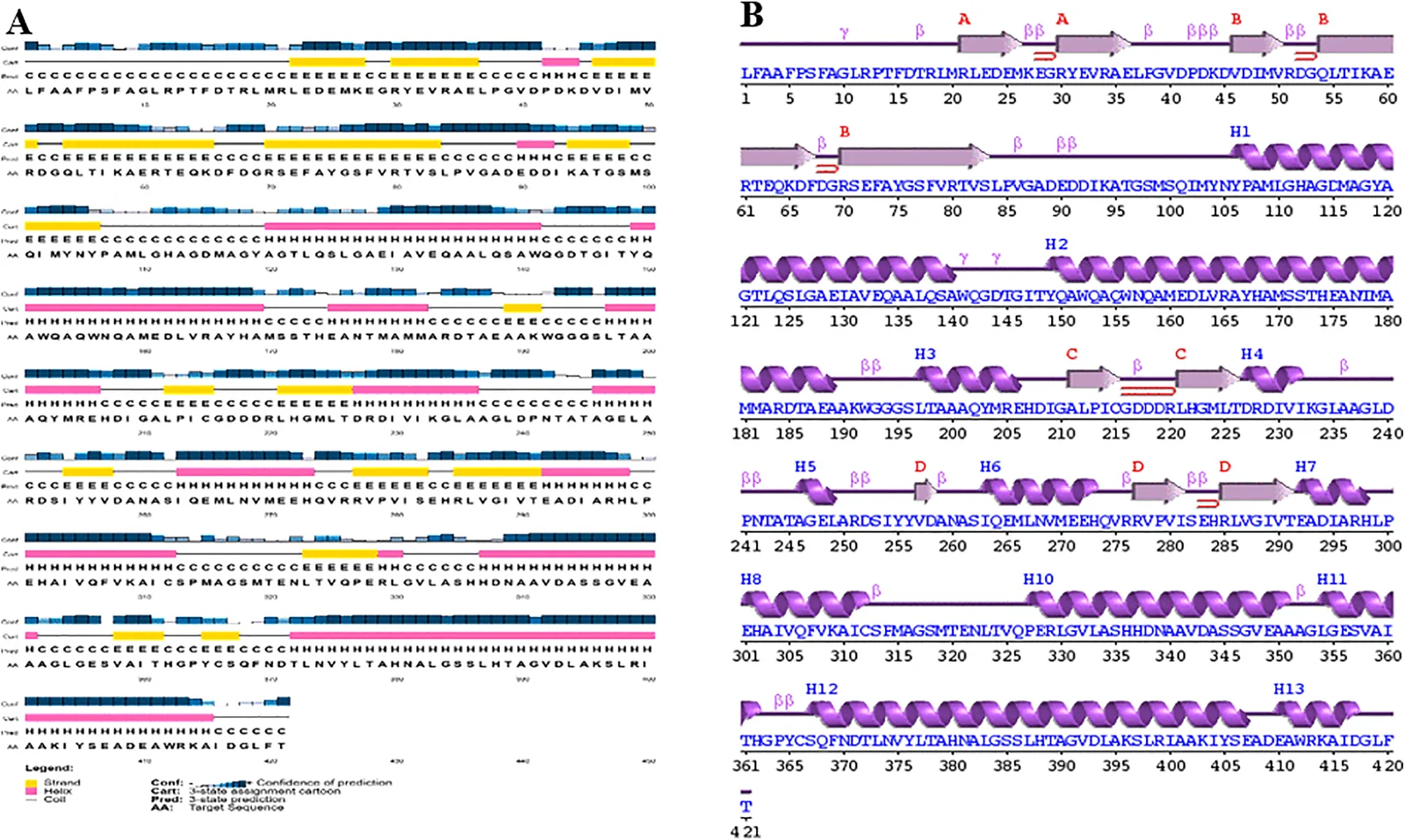
Secondary structure prediction. (**A**) The prediction of the 2D structure of the fusion construct as given by the PSIPRED tool. Helix, strands, and coils are shown in pink, yellow, and blue colors, respectively. (**B**) Secondary structure prediction using the PDBsum web server. Strands are represented by pink arrows (labelled by their sheets: A, B, C, D) and helices by purple springs (labelled as H1, H2...), while other structural motifs, β-turns and ɣ-turns, are also shown.

2D structure of the fusion protein, as generated by the PSIPRED tool, is shown in Fig 3A. It showed the presence of 41.09% *α-*helices, 23.5% β-sheets, and 35.39% coils, respectively. The 2D structure of the fusion protein was also predicted by the PDBsum web tool (Fig 3B). The tool predicted 4 sheets, 5 beta hairpins, 3 beta bulges, 1 beta-alpha-beta unit, 10 strands, 13 helices, 13 helices-helices interactions, 3 gamma turns, and 29 beta turns.

**Fig 4:**
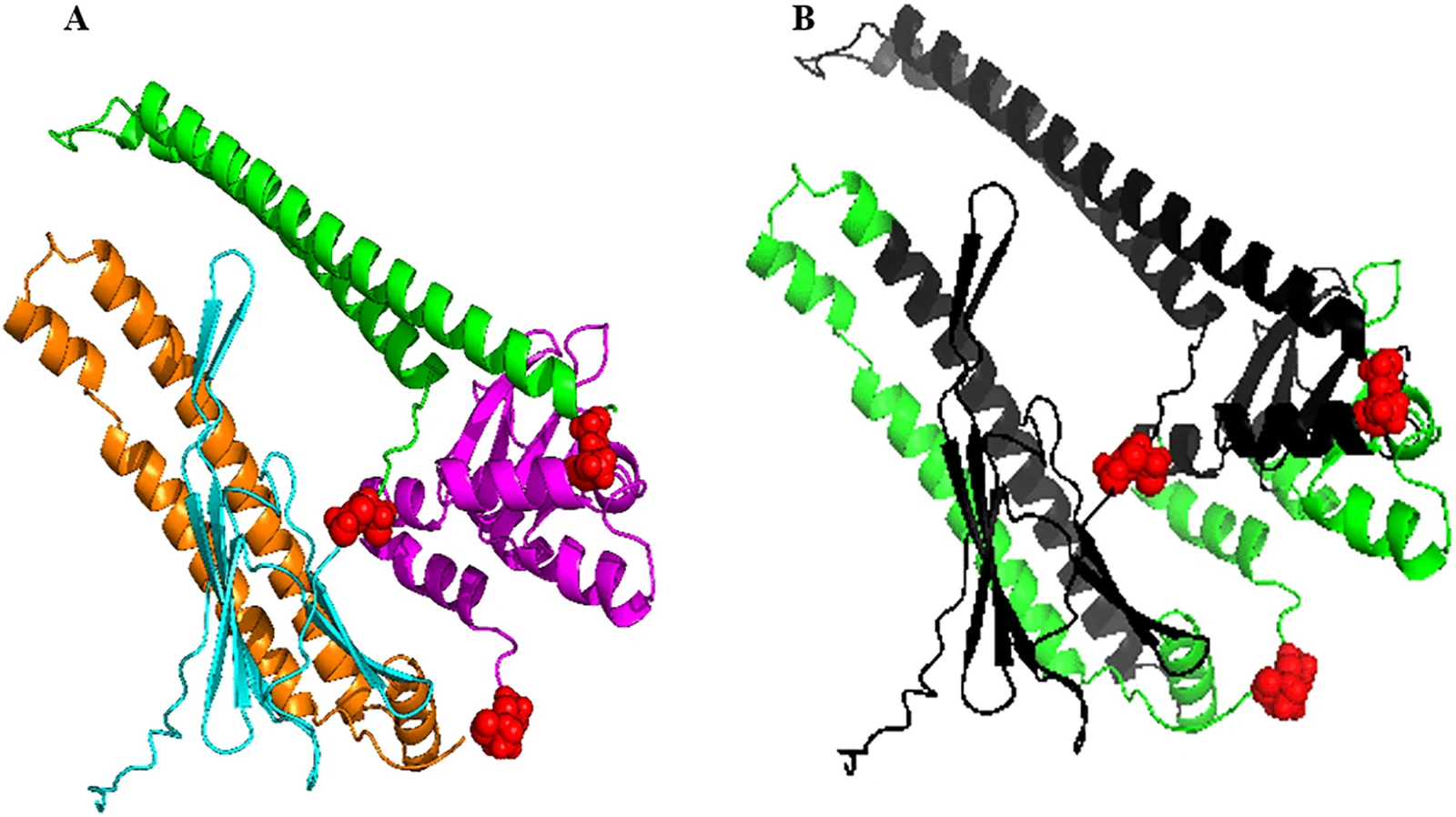
3D Structure of the fusion protein predicted by the AlphaFold2 server. (**A**) The component antigens, tnHspX, CFP7, EspC, and tnHrp1, are shown in cyan, orange, green, and magenta, respectively. (**B**) Immunodominant Th-1 cell epitopes and linkers are shown in black and red colors, respectively.

3D structure of the fusion protein predicted through AlphaFold2 is shown as Fig 4. All the contributing antigens to the fusion protein joined by -G-S- linkers are shown in different colors (Fig 4A). The position of epitopes predicted by PyMOL software is shown in black (Fig 4B). Most of the epitopic regions seem to be in a good accessible position.

### 3D Structure refinement and *in silico* prediction

**Fig 5:**
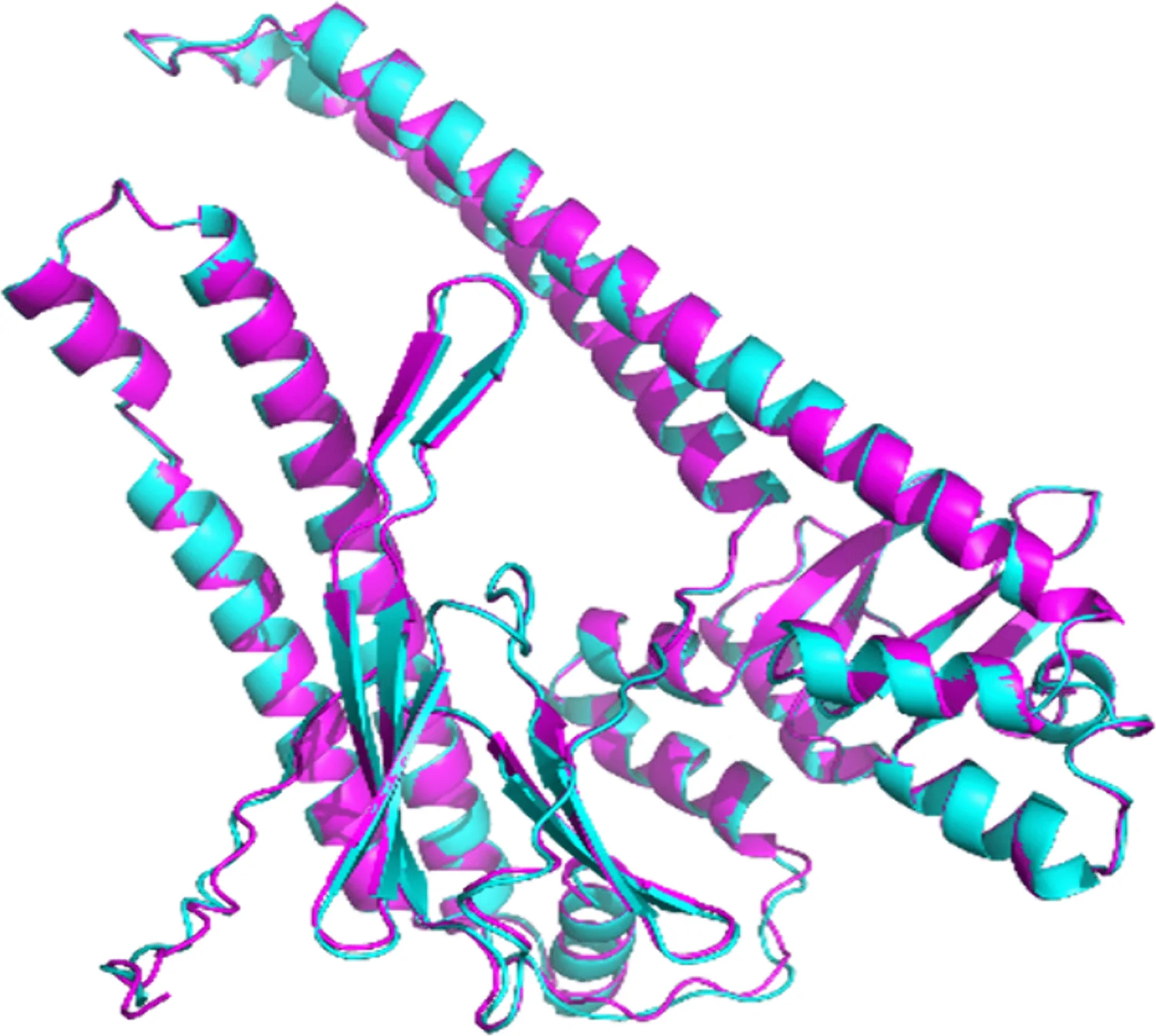
Structure refinement with GalaxyRefine web server. The refined fusion protein model (magenta) exhibits an improved stereochemical profile and limited steric clashes compared to the unrefined model (cyan).

The GalaxyRefine web server was employed to obtain improved stereochemical and energetic properties of the fusion protein. Amongst the five reformed models produced by this web server, MODEL 5 was predicted to be the most statistically favoured structure, with 99.3% residues in the most favoured region of the Ramachandran plot, with a clash score of 6.1 and a MolProbity score of 1.334 (Table 1). The refined model showed an RMSD value of 0.432 Å and a GDT-HA score of 0.9561, thus highlighting improved local geometry and greater structural similarity to the initial model. These results showed an improved structure prediction of the molecule. The 3D representation of the refined structure against the initial model is shown in Fig 5.

**Table 1:** Comparative analysis of the structure quality of the initial and refined protein model as generated by GalaxyRefine.

Model	GDT-HA	MolProbity	RMSD	Clash Score	Poor Rotamers	Ramachandran favoured (%)
Initial	1.0000	1.065	0.000	0.2	1.2	93.3
MODEL 1	0.9620	1.334	0.432	6.1	0.9	98.8
MODEL 2	0.9596	1.315	0.432	5.8	0.3	98.8
MODEL 3	0.9614	1.352	0.416	6.4	0.3	98.3
MODEL 4	0.9673	1.419	0.414	7.7	0.3	98.8
MODEL 5	0.9561	1.334	0.432	6.1	0.3	99.3

**Fig 6:**
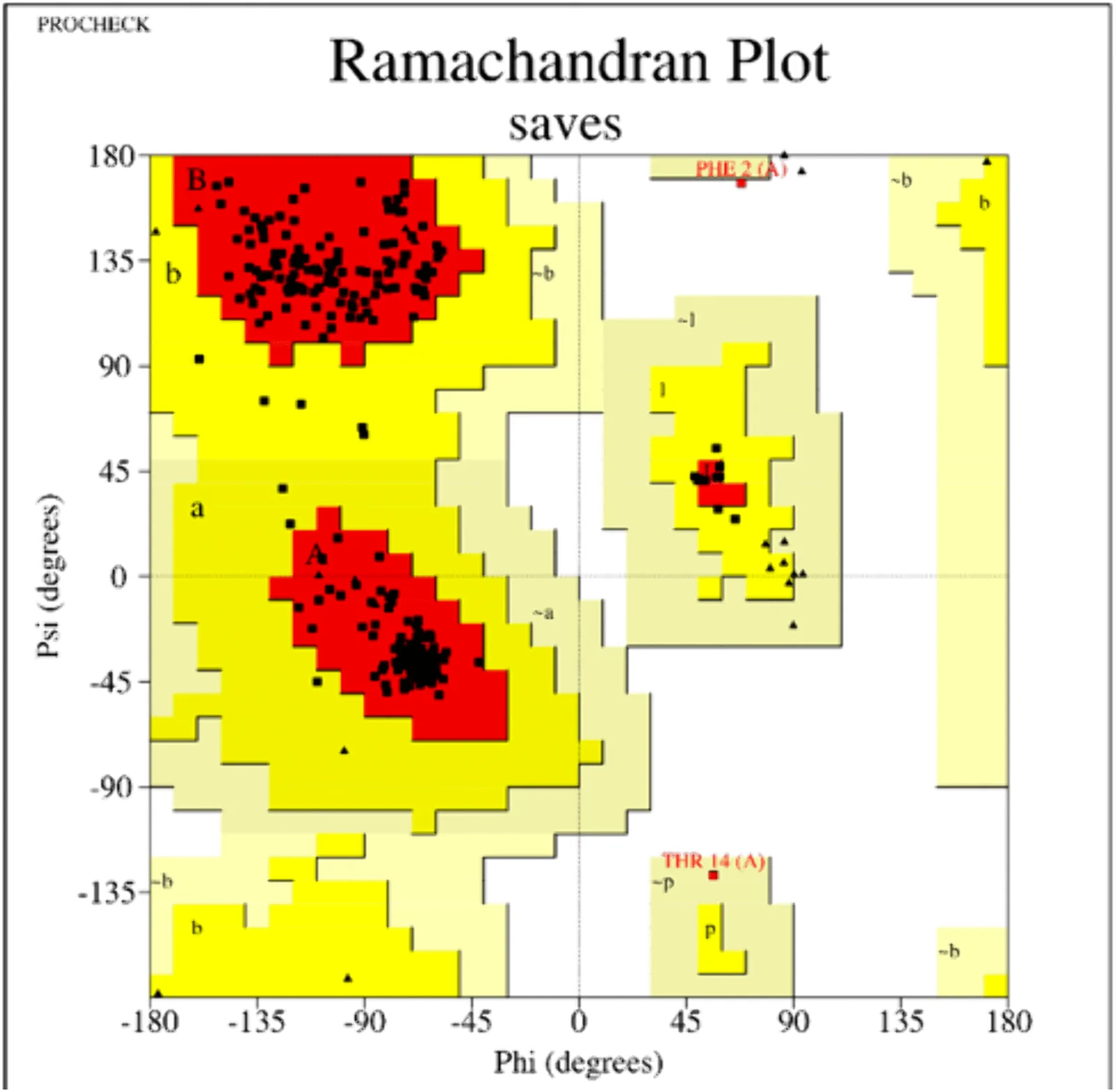
Ramachandran Plot generated by Procheck on SAVES v6.1. The plot statistics show the percentages of the favoured, additionally allowed, generally allowed, and disallowed residues as 96.0%, 3.5%, 0.3%, and 0.3%, respectively.

**Fig 7:**
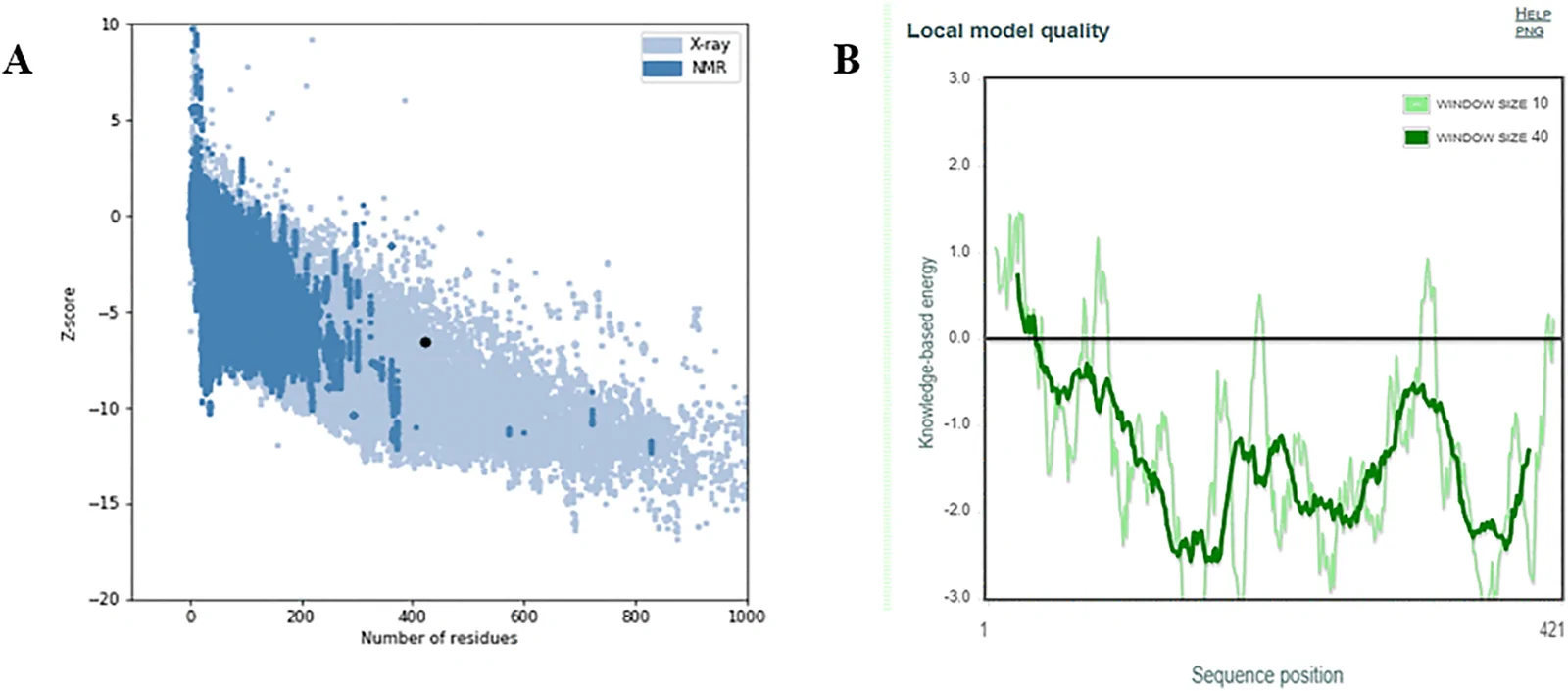
(A) Z-score plot generated by ProSA-web. The black dot for TetraFuVac11 shows a z-score of −6.8, which lies in the proximity of the z-score of the experimentally determined thermodynamically stable protein structures. (B) Local model quality of TetraFuVac11 with energy plotted as a function of the position of the residues in the protein sequence.

**Fig 8:**
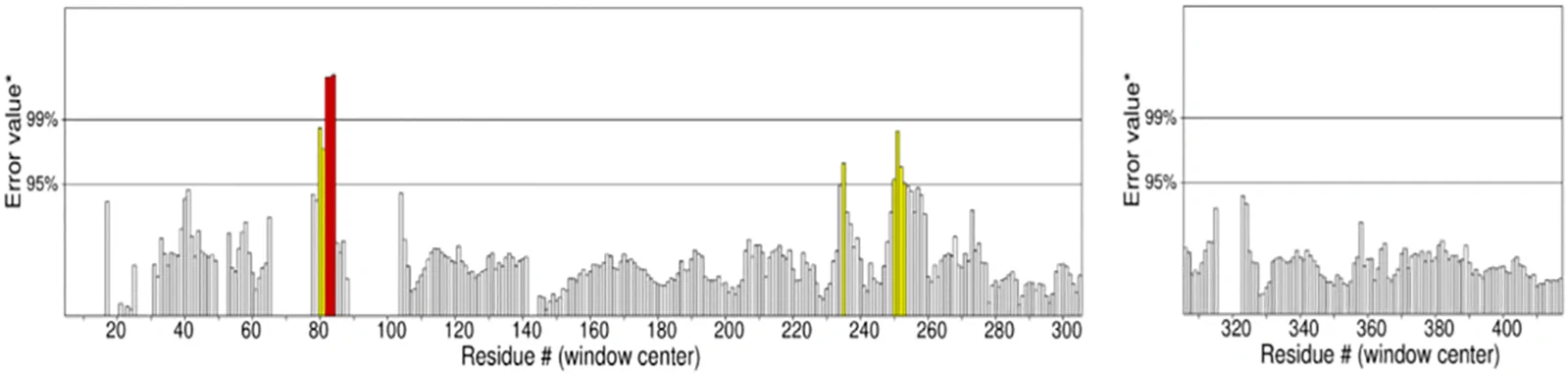
ERRAT plot generated by SAVES v6.1. Red bars indicate residues with an error value above 99%, yellow bars above 95%, and grey bars below the threshold value of 95%. The ERRAT score of the refined model was computed to be 97.1591.

The data generated by the Procheck-based Ramachandran plot showed 96.0% residues in the most favoured region, whereas the disallowed region contained only 0.3% (Fig 6). These statistics predict that the fusion construct has a stable and well-folded structure. The ProSA-web tool computed a Z-score of −6.8 for TetraFuVac11, which lies in the proximity of the Z-score of proteins with experimentally validated thermodynamically stable structures (Fig 7A). ProSA-web also showed a valid local model quality, and the energies of the majority of the residues in the fusion construct were negative (Fig 7B). The ERRAT score was 94.928 and 97.1591 for the unrefined and refined models, respectively. A large proportion of the residues were found below the rejection limit of 95 percent (Fig 8).

### Prediction of linear and discontinuous B-cell epitopes

The ABCpred tool predicted 14, 13, 17, and 12 B-cell epitopes for tnHspX, CFP7, tnHrp1, and EspC, respectively. For tnHspX, 10 epitopes were predicted to be non-allergenic, whereas all 14 were predicted as antigenic and non-toxic. Of the predicted 13 CFP7 epitopes, 7 were found to be antigenic and 8 non-allergenic, whereas all were predicted to be non-toxic. For tnHrp1, the numbers of antigenic and non-allergenic epitopes were predicted to be 5 and 8, respectively, and all were predicted to be non-toxic. Of the 12 predicted epitopes for EspC, 9 were identified as antigenic and 6 as non-allergenic, while all were predicted to be non-toxic. Overall, 10 for tnHspX, 4 for CFP7, 5 for EspC, and 2 for tnHrp1 were found to be antigenic, non-allergenic, and non-toxic. The complete data for the antigenicity, allergenicity, and toxicity evaluations of epitopes of all four antigens are provided in S6 Table. For the fusion protein, out of the total 66 predicted linear B-cell epitopes, 40 were antigenic, 37 were non-allergenic, and all were non-toxic (S6 Table). Overall, 25 of these epitopes were predicted as antigenic, non-allergenic, and non-toxic. Notably, the TetraFuVac11 construct possesses a greater number of predicted epitopes, suggesting that additional epitopes seem to be generated in a linker-mediated fusion molecule.

**Fig 9:**
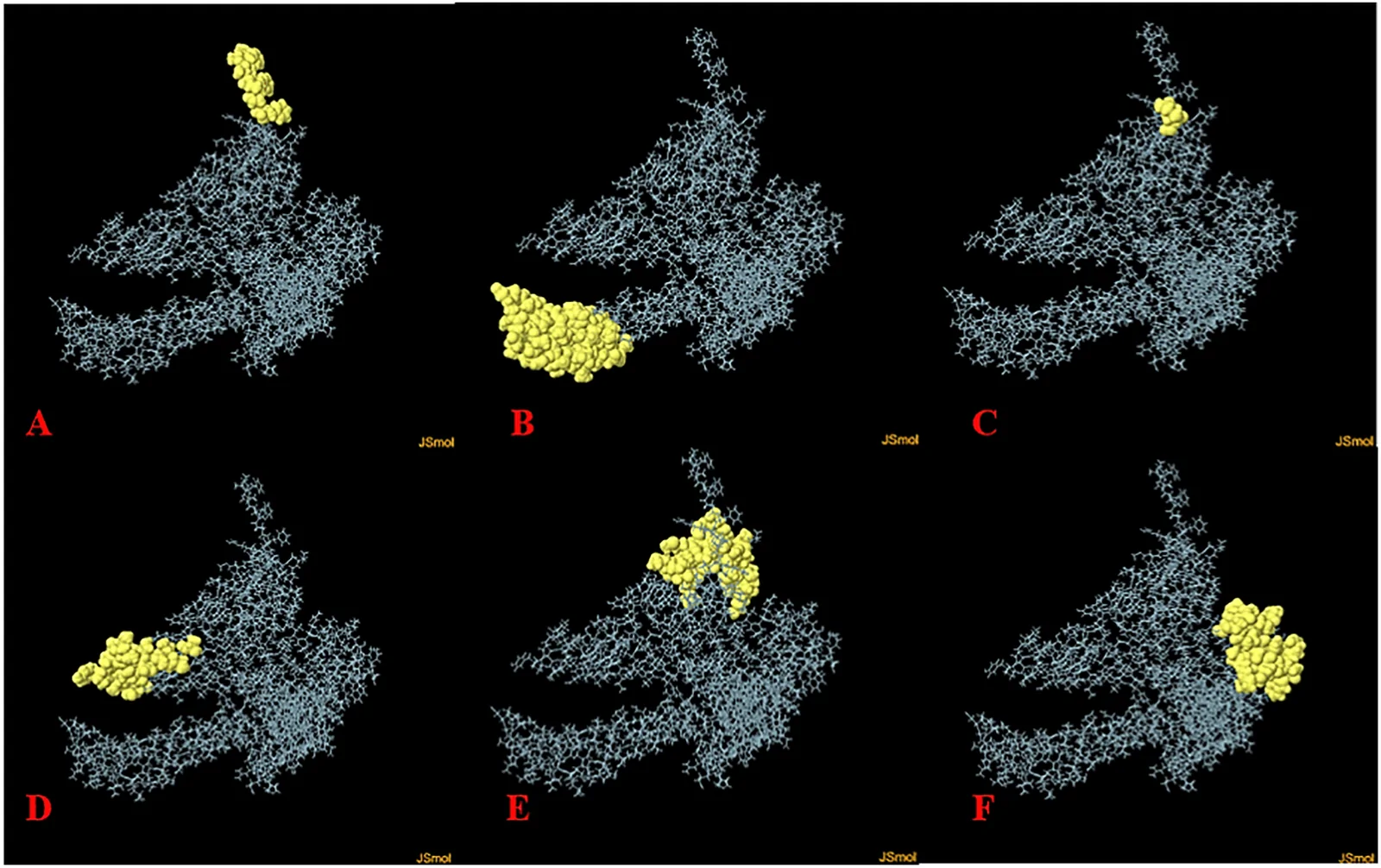
Spatial arrangement of discontinuous/conformational B-cell epitopes as predicted by the ElliPro web server. The fusion protein backbone is shown in grey sticks, while the predicted epitopes in all the above Figs (A-F) are shown as yellow spheres. Only epitopes with a score greater than 0.69 are shown.

**Fig 10:**
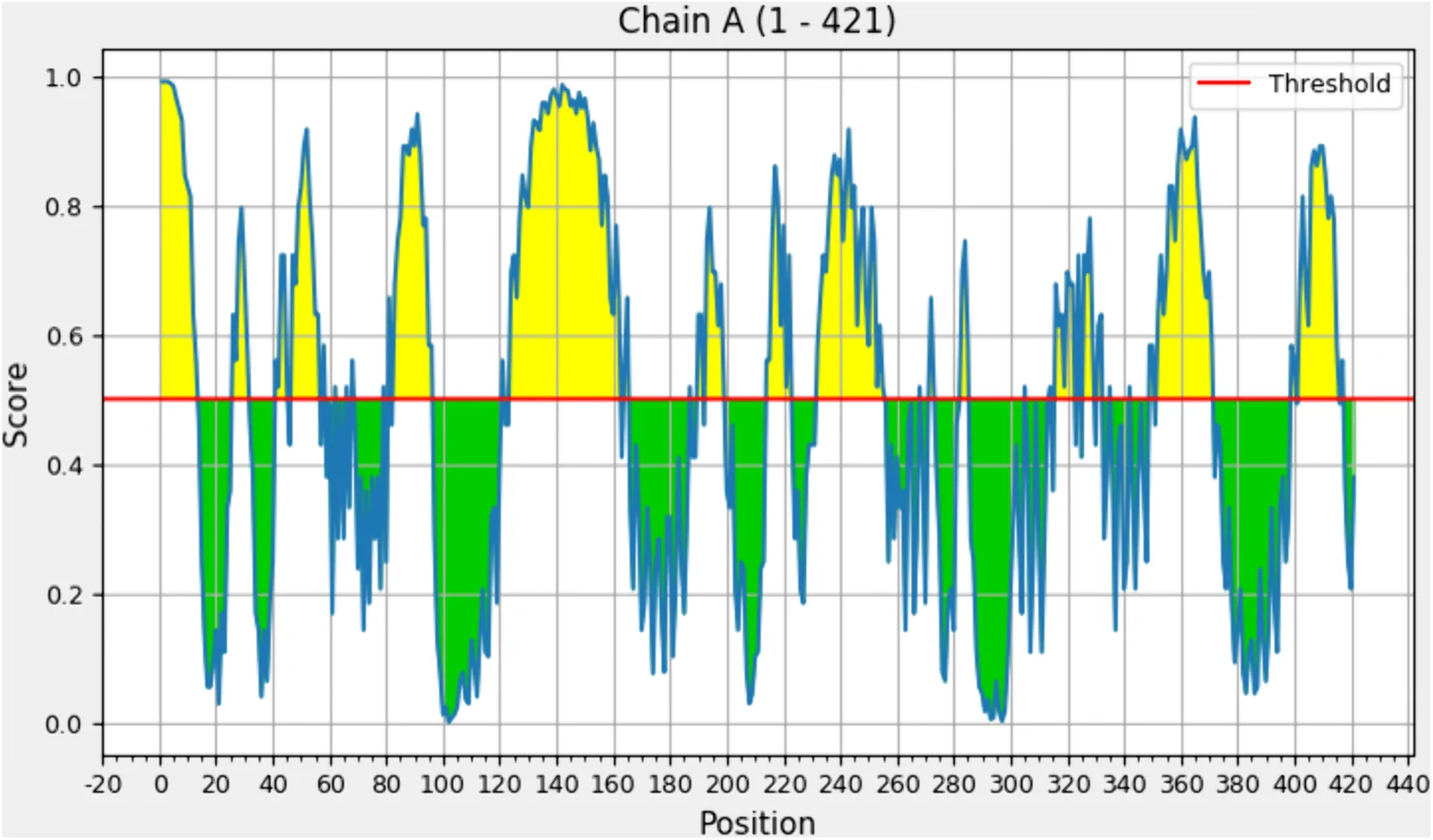
Individual scores of the predicted conformational/discontinuous B-cell epitopes of the fusion protein predicted by the ElliPro web server. The yellow colour represents potential B-cell epitopes with an epitope score above the threshold value of 0.5.

Ten discontinuous B-cell epitopes with PI values in the range of 0.546–0.976 were predicted for the fusion molecule by ElliPro software. The ten predicted epitopes with their respective scores are shown in Table 2. The predicted epitopes are represented by yellow spheres in the overall structure of the fusion protein backbone represented by grey sticks (Fig 9). ElliPro also generated a plot showing individual scores for the predicted conformational B-cell epitopes. In this plot, the yellow color represents epitopes with a confidence score above the 0.5 threshold, and the green color represents a moderate score (Fig 10).

**Table 2:** Discontinuous B-cell epitopes of the fusion protein predicted by the ElliPro web server, shown in descending order of their respective score. The location of the epitopes is shown as superscripts.

No	Residues	PI Score
1	L^1^FAAFPSF^8^	0.976
2	G^121^Q^124^S^125^L126GAEAVEQAALQSAWQGDTGITYQAWQAQWNQAM ED^162^, V^164^R^165^	0.854
3	A^9^GL^11^	0.831
4	A^350^A^352^GLGESVAITHGPYCSQFND^371^	0.771
5	M^26^K27E28G29R30Y^31^D^47^IMV^50^D^52^GQLT^56^K^58^V^79^T^81^S^83^, LPGADEDDIKAT^96^	0.738
6	E^321^NLTVQPER^329^A^402^KIYSEADEAWRK^414^ D^417^G^418^T^421^	0.697
7	A^187^A^190^ KWGGGSLTA^199^ I^214^CGDDDRLH^222^ I^232^ KGLAAGLDPNTATAGEL ARDSIY^255^ E^272^ H^273^ V^275^ E^283^ HR^285^	0.686
8	G^331^V^332^, S^335^, R^399^	0.588
9	V^40^DPDKD^45^	0.55
10	S^313^PMAGSM^319^	0.546

### Disulfide engineering of TetraFuVac11

**Fig 11:**
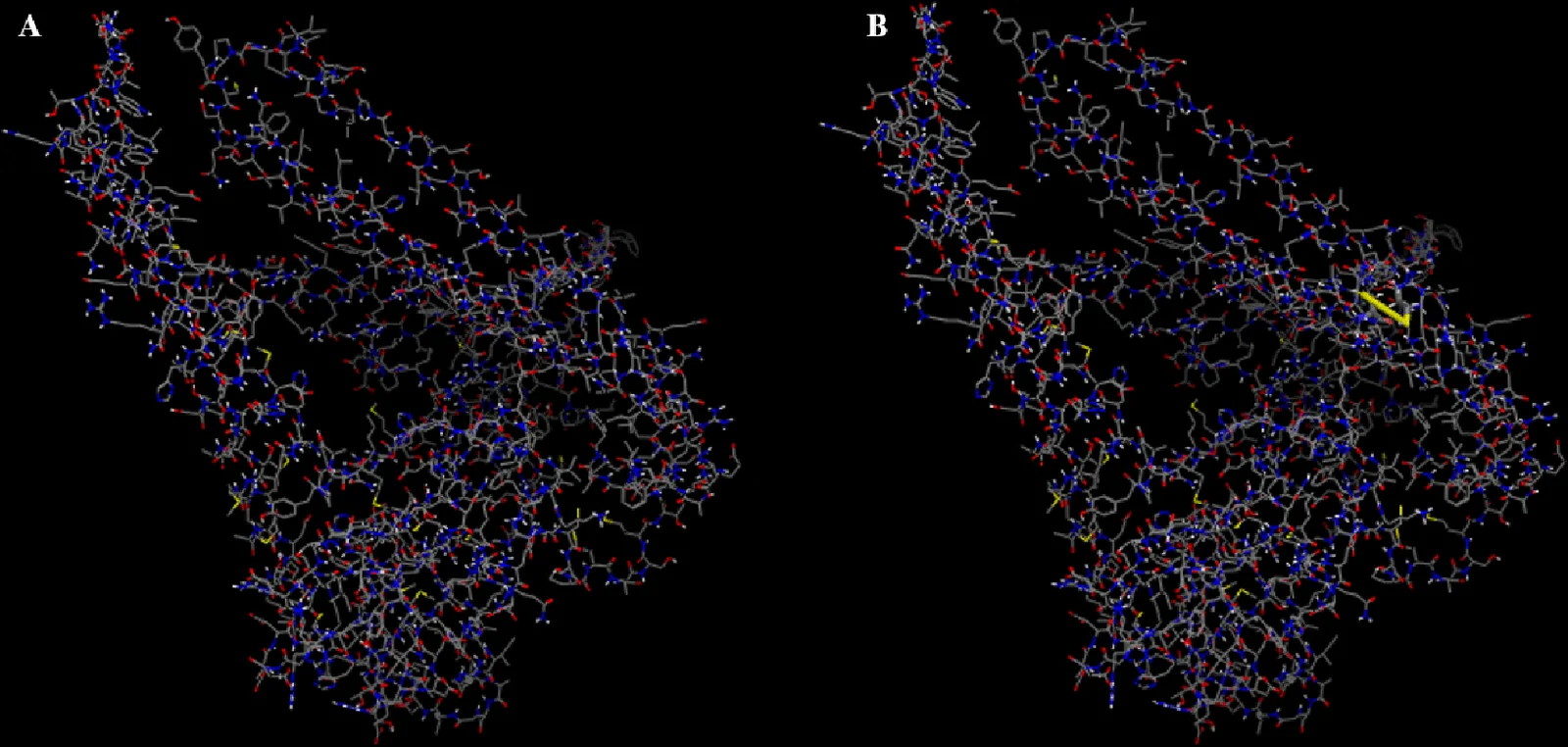
Disulfide engineering of the TetraFuVac11 refined structure. (**A**) Wild-type TetraFuVac11. (**B**) Mutant TetraFuVac11 with a disulfide bond shown in a yellow stick.

To improve the structural stability of the TetraFuVac11 vaccine construct, *in silico* disulfide engineering was executed using Disulfide by Design 2 v2.13 software. The software scanned 421 amino acid residues of the vaccine construct and identified a single residue pair, ALA334–ALA395 (1.27 kcal/mol), with bond energy <2.2 kcal/mol. The residue pair was mutated to cysteine to allow covalent cross-linking. Fig 11 shows disulfide engineering, in which the yellow stick represents a disulfide bond formed between cysteine 334 and cysteine 395.

### Molecular docking of TetraFuVac11 with TLR-4

**Fig 12:**
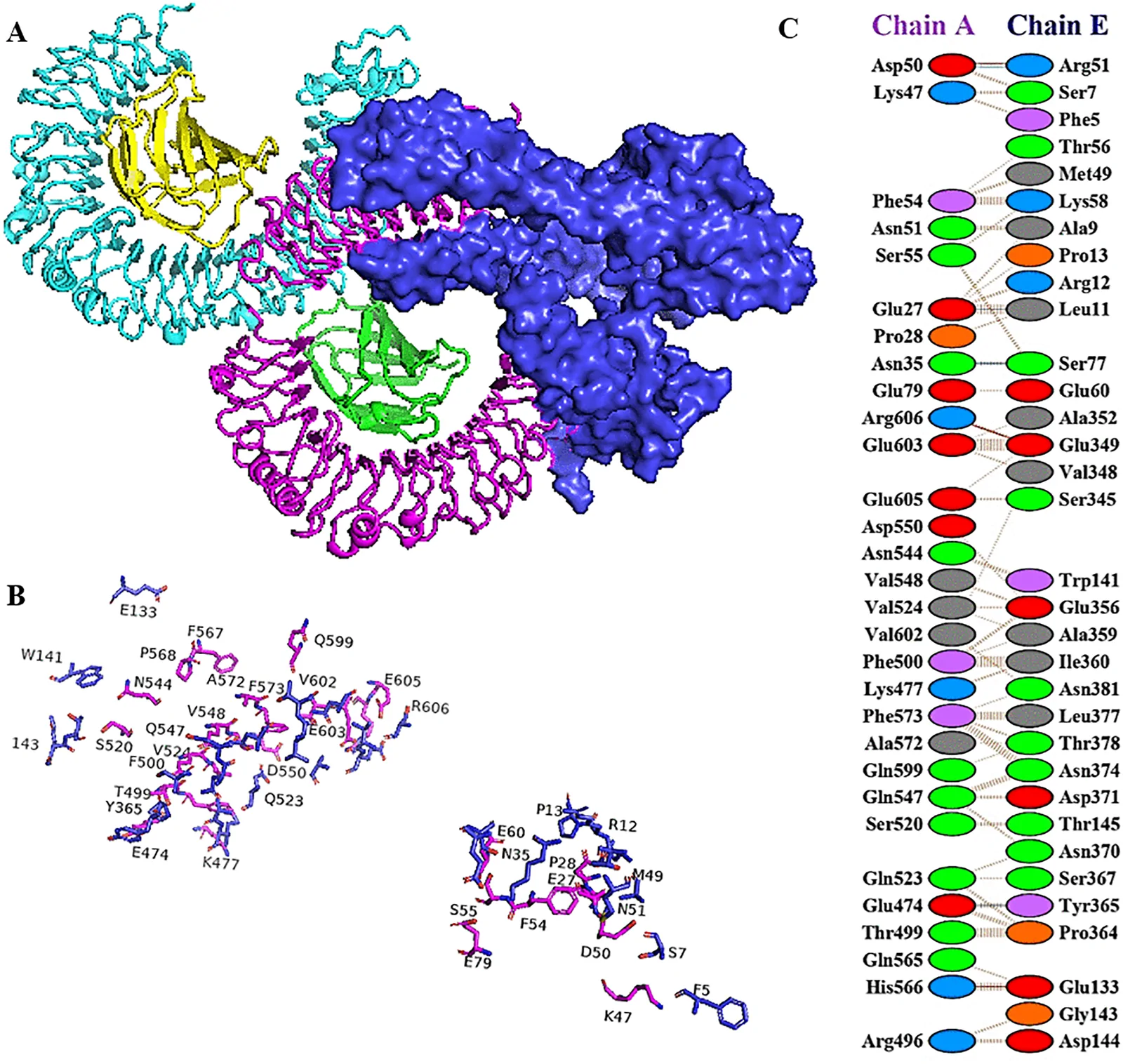
Docking analysis of the TetraFuVac11 construct with the TLR4 receptor. (**A**) Fusion-TLR4 docked complex generated through HDOCK, showing fusion protein in blue and chain A of TLR4 in magenta, chain B in cyan, chain C in green and chain D in yellow. (**B**) Interacting residues of the docked protein complex predicted through the PDBsum and visualized by the PyMOL web server. (**C**) The docking interactions obtained by PDBsum. Hydrogen bonds, salt bridges, and disulfide bonds are represented by blue, red, and yellow lines, respectively. The non-bonded interactions are denoted by orange arcs.

Molecular docking between the refined 3D model of TetraFuVac11 and TLR4 was conducted using the HDOCK server, which ranks protein complexes based on a hybrid method of *ab initio* and template-based docking. Out of the top 10 docked complexes (S7 Table) of the TetraFuVac11-TLR4 generated by HDOCK, MODEL-4 was considered to be the most favourable model based on a better initial thermodynamic profile as compared to the top 3 models (Fig 12A). The binding free energy was predicted to be −15.9 Kcal mol^-1^ by PRODIGY and −15.77 kcal mol^-1^ by HawkDock server. The dissociation constant for the docked complex was predicted to be 2.2 × 10^-12^ M by PRODIGY. PRODIGY also predicted several interfacial contacts (ICs) and non-interacting surfaces (NICs), as presented in Table 3. PDBsum showed that the fusion-TLR4 docked complex is stabilized by interactions between 33 amino acid residues from the fusion protein and 30 interacting residues from the receptor. Interacting residues of the docked protein complex were predicted through PDBsum and visualized by the PyMOL web server (Fig 12B). The formation of 239 non-bonded interactions, 3 salt bridges, and 4 hydrogen bonds contributed to the formation of the docked complex (Fig 12C).

**Table 3:** Docking analysis of the TetraFuVac11-TLR4 complex obtained from the PRODIGY web server.

Parameters studied	Values
Binding free energy (ΔG_bind_)	−15.9 kcal mol^-1^
Dissociation constant (Kd)	2.2 × 10^–12^ M
Interfacial Contacts (ICs)	11 Charged-charged, 16 charged-polar, 30 charged-apolar, 15 polar-polar, 40 polar-apolar, and 25 apolar-apolar.
Non-interacting Surface	NIS charged 24.32%

### Molecular dynamics simulations and normal mode analysis

**Fig 13:**
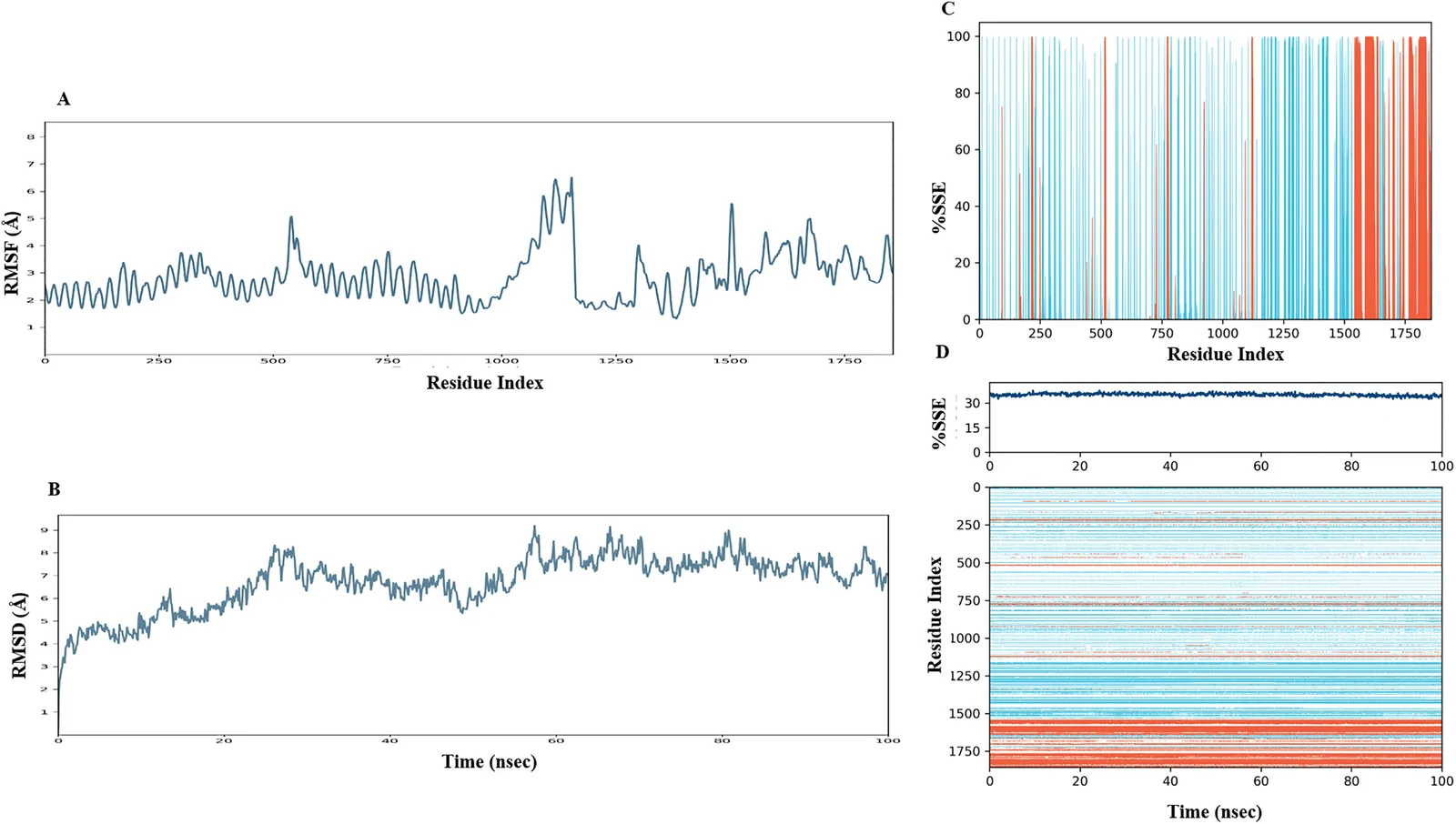
Molecular dynamics simulations of the TetraFuVac11-TLR4 complex. (**A**) RMSF plot. (**B**) RMSD plot. (**C)** SSE distribution by residue index showing beta-strands in cyan and α-helices in orange. (**D**) SSE composition of each frame across the simulated trajectory.

**Fig 14:**
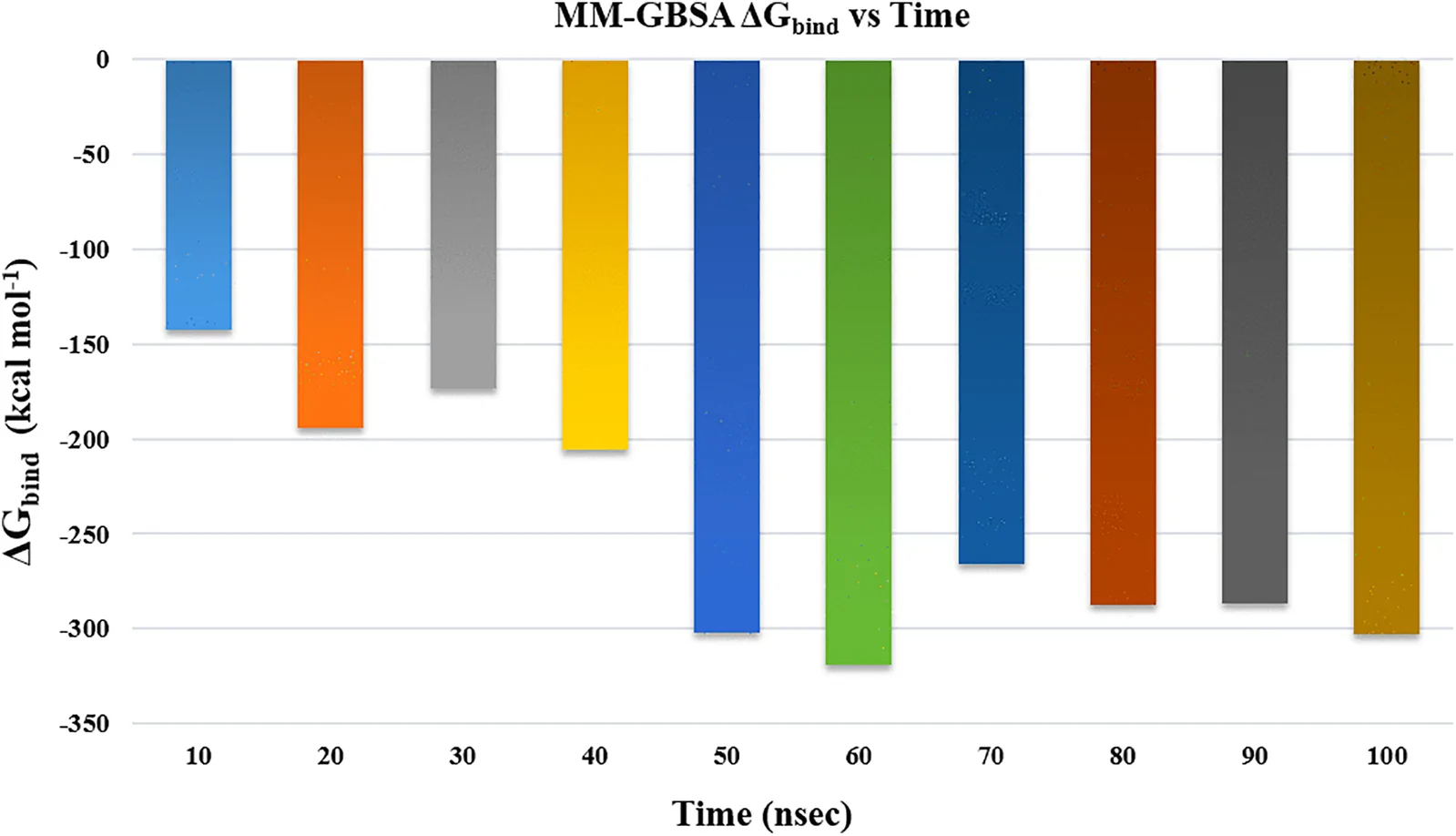
The binding energy (ΔG_bind_) of the TetraFuVac11-TLR4 docked complex, as calculated by the MM-GBSA approach across different frames of the 100 ns simulation.

The RMSF plot demonstrated the local flexibility of the TetraFuVac11-TLR4-docked complex over 100 ns. A large majority of the TLR4 residues exhibited low-level fluctuations (~2–3 Å) except for a larger peak reaching 6.5 Å around ~1100–1160 residue indices. As the RMSF plot transitioned towards the fusion protein, the baseline drifted slightly upward, showing moderate fluctuations and some elevated peaks, which is a typical trend for a linker-mediated fusion protein (Fig 13A). In the RMSD plot, the system initially deviated from ~2 Å to ~8 Å until 60 ns as the protein underwent conformational relaxation. After approximately 60 ns, the docked complex was equilibrated, and the RMSD fluctuated around an average value of ~7–8 Å without sustained drift, suggesting that the docked protein complex has attained a relatively stable conformational ensemble distinct from the initial structure (Fig 13B). As the docked complex had five chains (chains A-D for TLR4 and TetraFuVac11 in chain E), the RMSD values were slightly higher. The conservation of various secondary structural elements in the docked complex was studied through SSE plots as a function of residue index and simulation time (Fig 13C, 13D). The TLR4 receptor is dominated by beta-strands (cyan), and the fusion protein by α-helices (orange). The docked complex showed a 35.15% mean total SSE, with 13.35% α-helices and 21.80% strands (Fig 13C). The composition of secondary structure elements remained conserved over different frames of 100 ns of simulation, showing minor fluctuations between 33% and 36% (Fig 13D). The binding free energy (ΔG_bind_) of the docked molecule was estimated across selected frames from the 100 ns of simulation. The net total binding free energy (ΔG_bind_), as predicted by the MM-GBSA approach, was found to be −248.1 kcal mol ^-1^. Besides, van der Waals interactions (−375.51 kcal mol^-1^) significantly contributed to the overall stability of the docked complex. The binding energy of the docked complex across 10 evenly spaced frames for a 100 ns simulation is shown in Fig 14.

**Fig 15:**
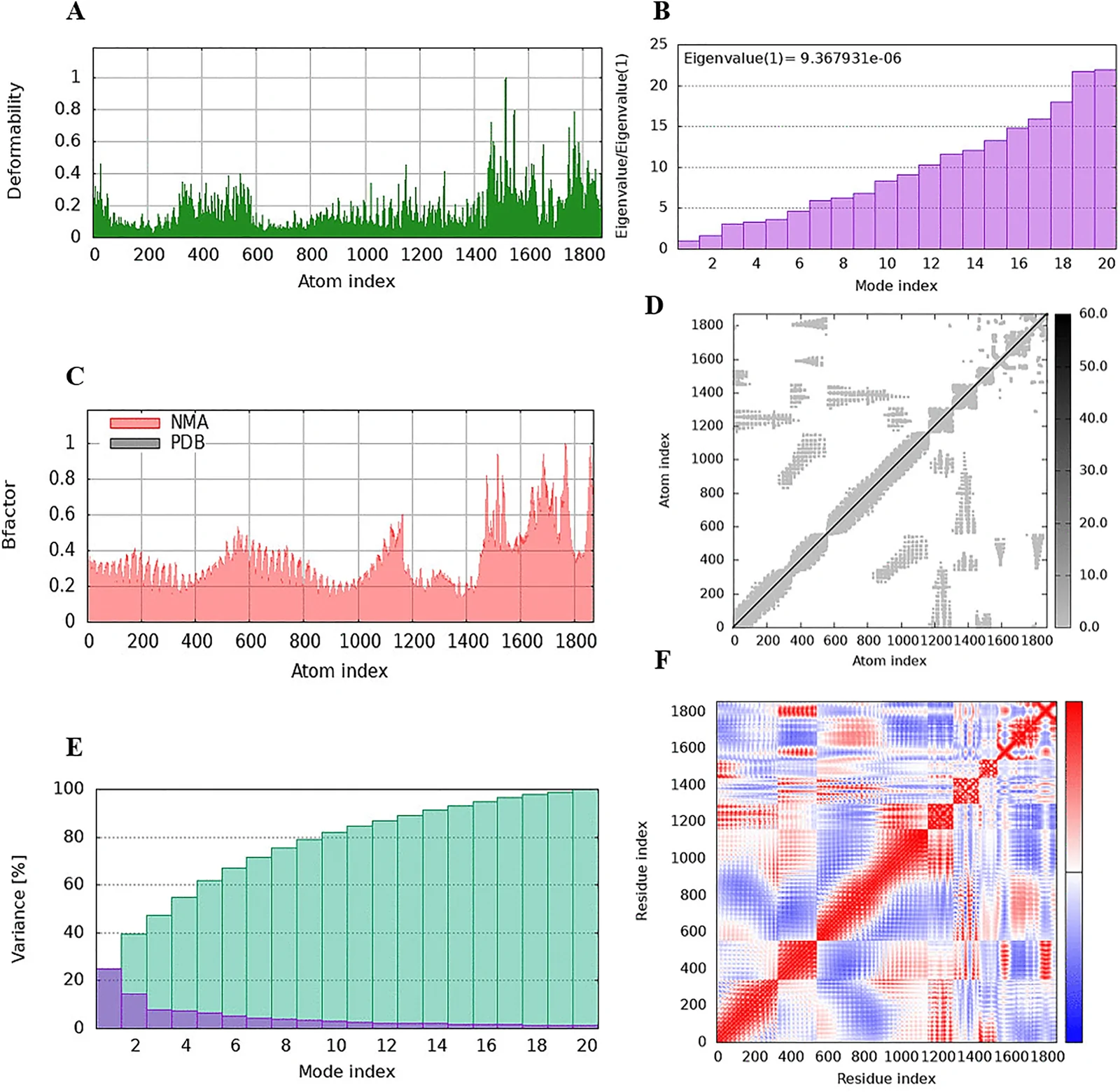
Normal mode analysis of the TetraFuVac11-TLR4 docked complex as generated through the iMODS web server. (**A**) The deformity plot shows stable regions in the form of valleys and the flexibility levels in the form of peaks. (**B**) An Eigenvalue (1) of 9.367931 × 10^−6^, which represents the ease with which a particular structure. **(C)** The B-factor mobility plot showing limited atomic fluctuations across the majority of the residues. (**D**) Elastic network model showing stiffer regions in dark grey spots. (**E**) The variance plot showing cumulative variance in green and individual variance in purple. (**F**) The covariance plot indicating the correlated, anticorrelated, and uncorrelated motion of the residues in red, blue, and white, respectively.

Additionally, Normal Mode Analysis (NMA) was performed using iMODS. The deformity plot showed that most residues are distributed in the valleys, with low-amplitude minor peaks distributed across the outer loop of the TetraFuVac11-TLR4 docked complex (Fig 15A). An Eigenvalue (1) of 9.367931 × 10^−6^ was obtained for the lowest mode (Fig 15B). The B-factor mobility plot showed that most residues exhibit minimal B-factor between 0.2 and 0.4. However, minor peaks are seen across the atomic index 1450–1860 (Fig 15C). In the elastic network model, the stiffer regions are shown in dark grey, corresponding to structural rigidity within the TetraFuVac11-TLR4 docked complex (Fig 15D). In the variance plot, cumulative and individual variance are represented in green and purple colors, respectively (Fig 15E). The individual variance shown in purple gradually decreases as the mode index increases. The first few modes contribute to the overall variance. In the covariance plot, the blue regions represent residues that move in opposite directions, while the red and white regions depict correlated and unrelated motion, respectively. Along the main diagonal, the clustering of dark red blocks indicates the synchronized structural domain mobility within the TetraFuVac11-TLR4 docked complex. Similarly, synchronized long-range correlated movements are apparent from the distribution of dark red blocks away from the diagonal axis (Fig 15F).

### Immune simulations

**Fig 16:**
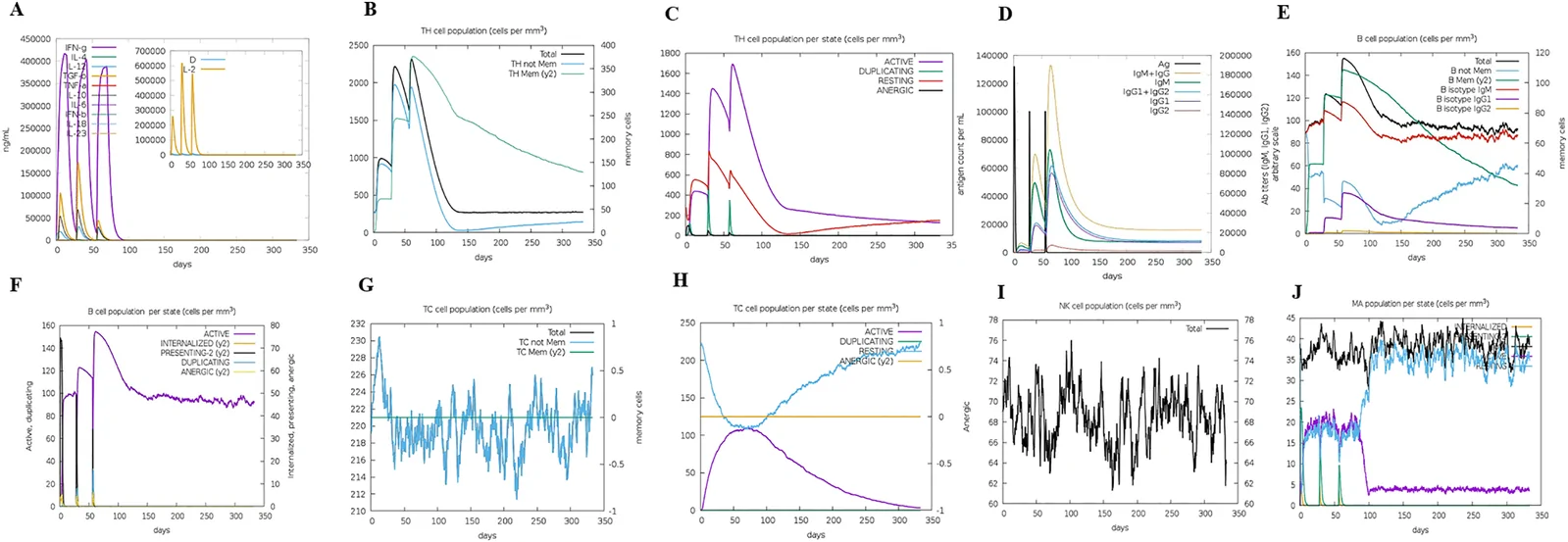
Immune simulations using the C-ImmSim web server for 1000 simulation steps. (**A**) Cytokine concentration profile showing a rapid surge in IFN-ɣ as a primary immune response, followed by the activation of IL-2, IL-10, and IL-12. The danger signal is represented by D in the inset plot. **(B)** TH cell population. **(C)** TH cell population per state. **(D)** Level of antibody and antigen. **(E)** B cell population. **(F)** B cell population per state. **(G)** TC cell population. **(H)** TC cell population per state. **(I)** NK cell population. **(J)** The Macrophage cell population.

The C-ImmSim web server predicted a durable immune response against the designed fusion protein molecule. Three distinct peaks for three different times of injection were seen in the cytokine profile. A rapid release of IFN-ɣ (violet/purple) during each injection highlighted the activation of a strong primary immune response. The peak showing highest level of IFN-ɣ (around 42 × 10^5^ ng/mL) was seen after the first dose. Followed by IFN-ɣ, a rapid but comparatively transient activation of IL-2 (bright yellow) was seen in the insert plot. A low amplitude and broad peak was seen for IL-10 (black) as the simulation progressed (Fig 16A). The primary dose initiates the production of the TH cell population, which keeps on increasing following booster doses (Fig 16B).

Along with cytokine kinetics, C-ImmSim provided thorough information about the transition between different functional states of the T-cell population following vaccine administration. As shown in Fig 16C, a rapid surge in the active (purple) TH cell population was seen following each injection. The population of duplicating TH cells (green) increased steadily in the beginning and reached its maximum level around the 30th to 35th day after the injection, supporting clonal expansion. A progressive rise in the population of resting TH cells (red), especially after booster doses, indicates progression to the memory cell stage. The immune tolerance was persistent at the lowest levels during the whole process, as indicated by the limited activation of the anergic TH-cell population during the whole process. The primary dose sharply increased the antigen level, followed by a decline in the subsequent doses. On the other hand, the booster doses significantly increased the antibody level compared to the primary dose (Fig 16D). Following each booster dose, memory B cells and specific B cell isotypes significantly increased. The increase in IgM level was more pronounced compared to IgG2 (Fig 16E). A rapid burst in the active and duplicating B-cell population was seen following each dose, and their level declined quickly after clearance of the antigen (Fig 16F). In the case of cytotoxic T-cells, noisy fluctuations were seen around a stable baseline, with a sharp peak showing an increased level of active cytotoxic T-cells around day 60 (Fig 16G and 16H). The natural killer cell population exhibited a fluctuating trend after each dose of infection (Fig 16I). During the initial days (0–90), an elevated level of active macrophages was seen while the resting population remained low. After day 100, the resting macrophage population increased and reached a stable level, but the active macrophage population dropped to the minimum baseline level (Fig 16J).

### Prediction of surface accessibility

**Fig 17:**
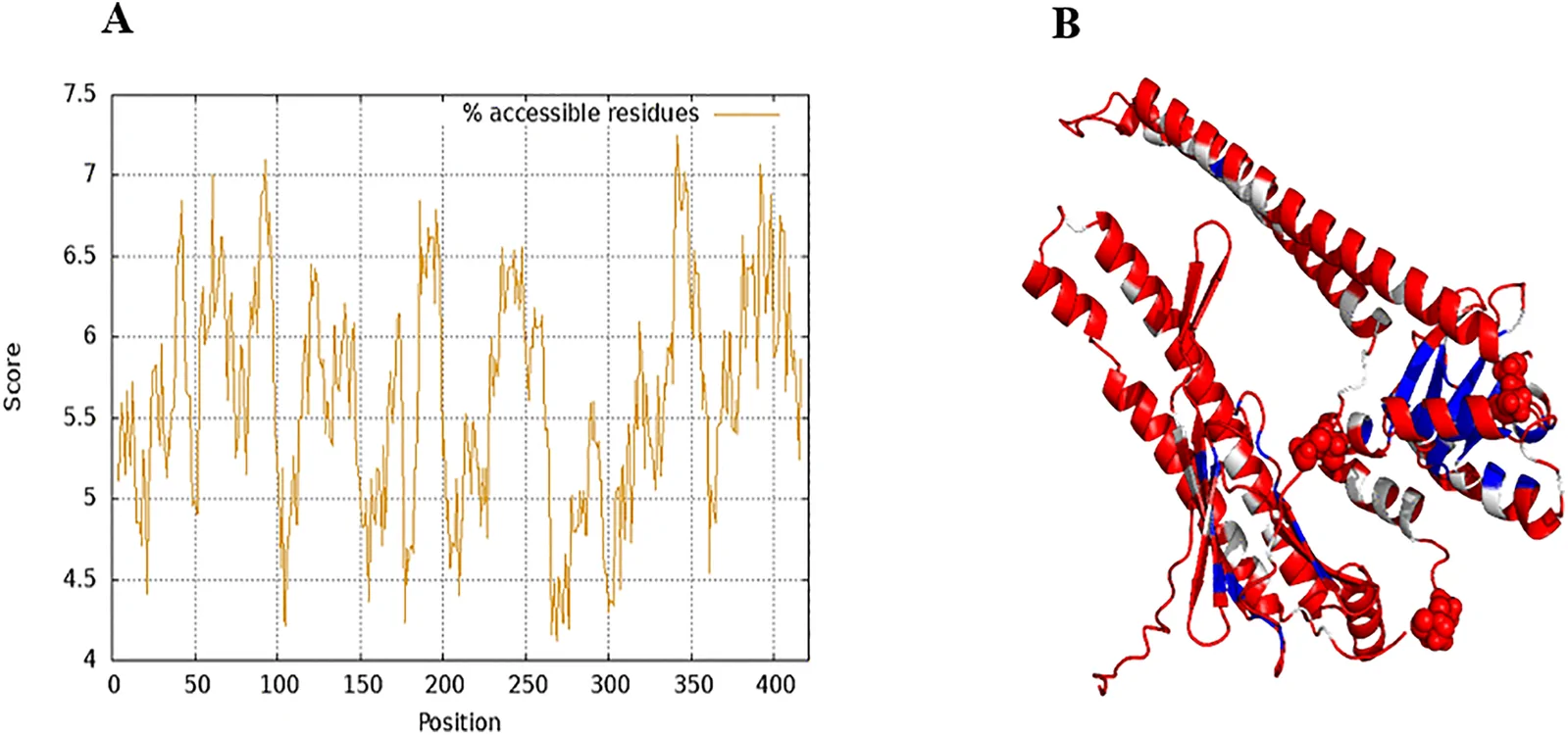
Prediction of surface accessibility. (**A**) ProtScale analysis of the fusion protein, showing % accessibility of the residues in the form of peaks. Most of the residues lying in the stretches 39-44, 53-72, 84-97, 116-124, 134-142, 185-199, 234-260, 336-356, and 380-412 show a high score (> 6). The maximum peak is seen at the 341 residue position. (**B**) NetSurF analysis of the fusion protein. The red color highlights surface-exposed residues (68.41%), the blue color shows buried residues (31.59%), whereas the white color shows partly exposed residues with an intermediate RSA value.

The ProtScale web server showed several probable surface-accessible regions in the fusion protein sequence. The highest peak was observed at amino acid position 341, with an accessibility score of 7.244. The other major peaks were seen at the 39–44, 53–72, 84–97, 116–124, 134–142, 185–199, 234–260, 336–356, and 380–412 stretches of amino acids. The majority of residues in these stretches were predicted to have an accessibility score greater than 6 (Fig 17A). NetSurfP, the other tool used for the prediction of surface accessibility, showed that the fusion protein is dominated up to the extent of 68.41% by surface-exposed residues with an appreciable RSA value, while 31.59% of residues were found to be buried (Fig 17B).

### *In silico* cloning and solubility prediction

**Fig 18:**
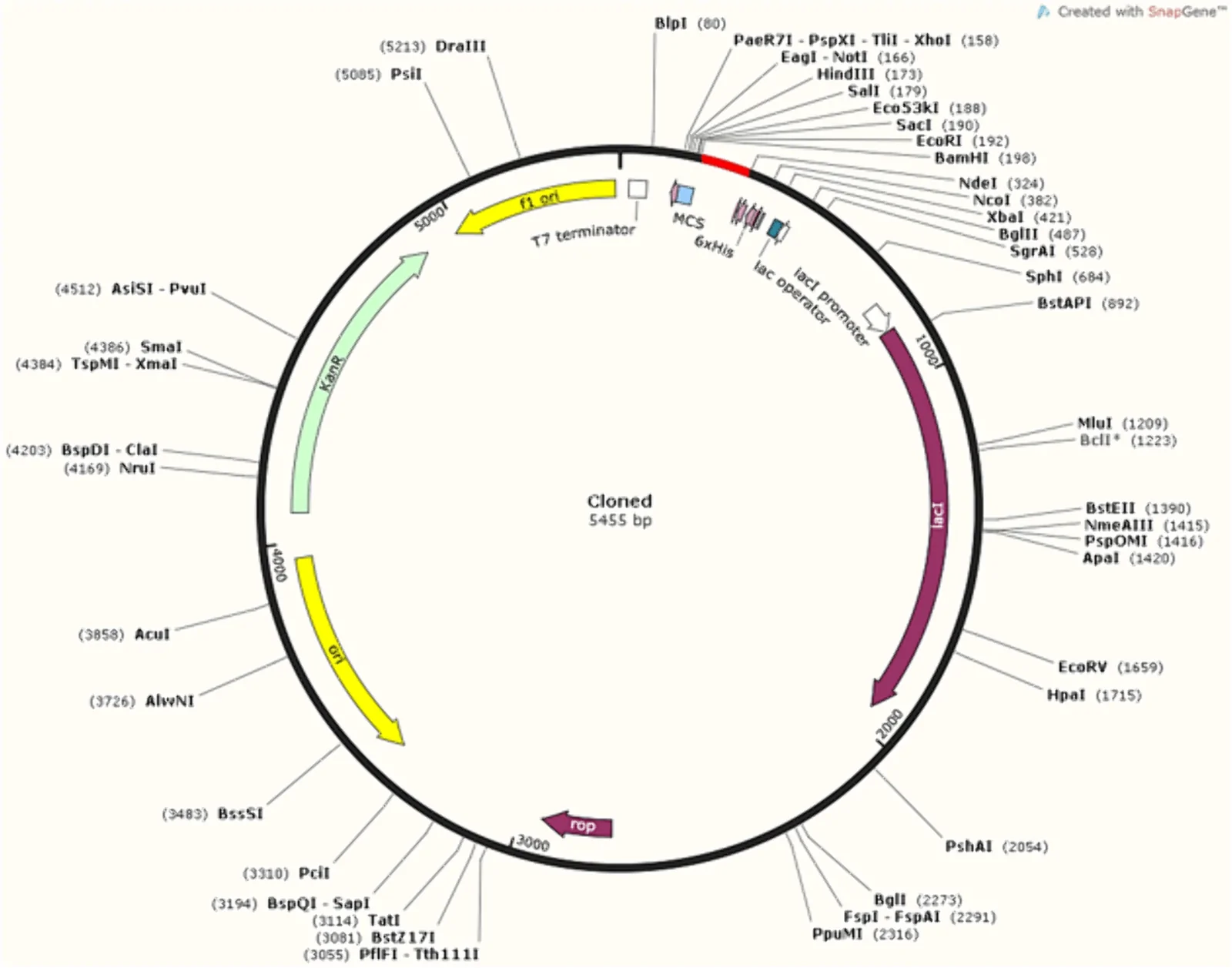
Recombinant pET-28a(+) vector generated through SnapGene software. In silico restriction cloning of TetraFuVac11 in the pET-28a(+) vector between NdeI and BamHI restriction sites. The vector backbone is shown in black, whereas the cloned fragment is depicted in red.

The recombinant pET-28a(+) vector containing TetraFuVac11 was generated using the SnapGene software tool. The fusion molecule, after codon optimization, was integrated between the *Nde*I and *Bam*HI restriction sites of the vector. The resulting clone is shown in Fig 18.

**Fig 19:**
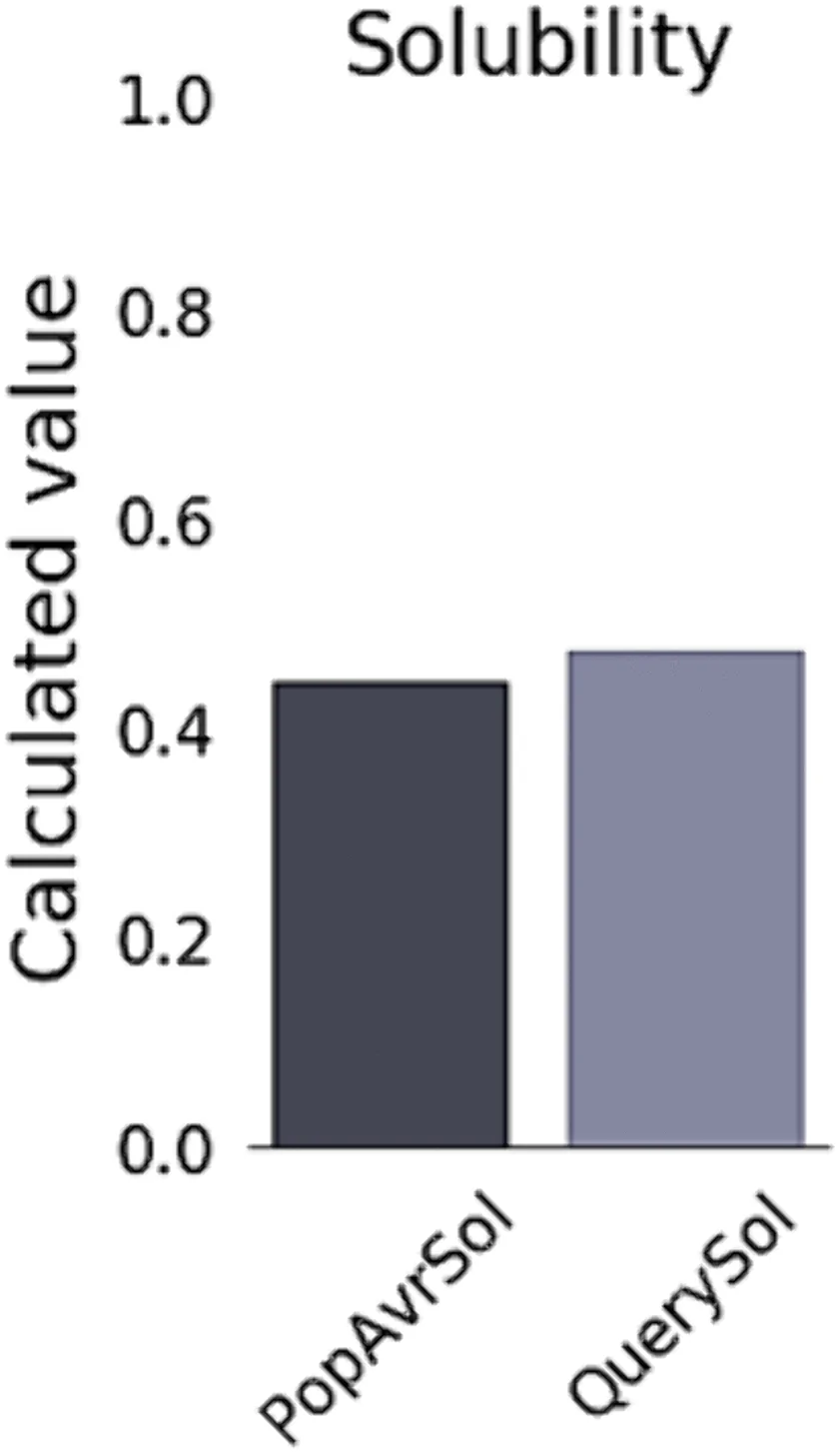
Solubility prediction by ProteinSol server. The predicted solubility for the fusion protein is 0.47.

Using the SoluProt 1.0 software, the solubility score for the TetraFuVac11 molecule was predicted to be 0.91, which is substantially higher than the 0.5 threshold. Using ProteinSol, the solubility score was predicted to be 0.47, exceeding the average solubility score of 0.45 for the proteins expressed in *E. coli*. (Fig 19).

## Discussion

A vaccine for prophylactic and therapeutic applications against tuberculosis caused by *Mtb* is essential for effectively controlling the disease. In recent years, reverse vaccinology has become a common approach for vaccine development against different diseases [65]. The selection of antigens based on predicting immunogenic epitopes using *in silico* tools is crucial to vaccine development. The selection of antigens with T-cell-specific epitopes is important, as they promote activation of adaptive immunity against invading pathogens [66]. This study involved an in-depth and systematic *in silico* analysis to construct a fusion molecule comprising HspX, CFP7, EspC, and Hrp1 antigens. These antigens were chosen based on their specific availability at different stages, like latent, early, and active states of the disease. Of the antigens used, HspX and Hrp1 are specific to the latent stage, CFP7 to early stage, and EspC to the active stage of the disease [9,15,67]. Moreover, all the selected antigens are reported to have epitopes specific to both T and B cells, which play a critical role in the development of an effective vaccine, as reported previously [68]. The antigens, HspX and Hrp1, were used in the truncated form by deleting fragments at their N- and C-termini to remove their non-epitopic fragments. The removal of non-epitopic segments greatly improves the surface accessibility of the epitopes, as reported previously for other *Mtb* antigens [33,69]. However, CFP7 and EspC were used without truncation, as their epitopes are known to be dispersed over the entire sequence. The four components were joined together through flexible -G-S- linkers, and the overall size of the fusion molecule is within a good working range for expression in *E. coli* and subsequent processing. -G-S- linkers are likely to prevent inter-domain interactions, promoting the possibility of each domain to fold independently of the others into a biologically functional conformation by maximizing rotational freedom and reducing steric clashes [70].

While some individual epitopes for the component antigens of the fusion protein were predicted to be non-antigenic and allergenic but the fusion molecule, as a whole, was identified as antigenic, non-allergenic and non-toxic. The acidic nature of the fusion protein, as predicted by the ProtParam web server, was indicated by a pI value of 4.79. The hydrophilic nature of the fusion protein was also predicted by a GRAVY value of −0.182. The hydrophilicity property of TetraFuVac11 reinforces the potential of soluble expression in *E. coli*. The secondary structure prediction by the sequence-based tool PSIPRED and the structure-based tool PDBsum identified 13 helices in the TetraFuVac11 structure. However, a variable number of strands and coils was predicted by the two tools. As reported previously, due to inherent algorithmic constraints, PSIPRED provides more accurate prediction (77% accuracy) for helices compared to β strands (70% accuracy) [71].

The GalaxyRefine web tool significantly refined the structure of the 3D model of the fusion protein generated through AlphaFold2, which is reflected in the increased percentage of most favoured residues from 93.3% to 99.3% for the refined model (Table 1). However, the Procheck web server showed 96.0% most favoured residues in the Ramachandran plot. The Ramachandran plot statistics generated with the Procheck web server are different from those generated by GalaxyRefine. The reason for this variation is that both tools use different algorithms for making a Ramachandran plot [72]. Overall, these values for Ramachandran statistics suggest that the fusion protein TetraFuVac11 possesses a stable structural conformation. Similarly, the ERRAT score of 94.928 for the unrefined model improved to 97.1591 for the refined model, thus suggesting a higher level of accuracy for the refined model of the fusion molecule.

Whereas the majority of linear epitopes predicted by the ABCpred tool for each of the component antigens are reflected in the fusion molecule, a few additional epitopes also appear, which seem to be due to the addition of linkers between the antigens. The Ellipro web server generated discontinuous/conformational B-cell epitopes with 0.546 to 0.976 PI scores. Six of these epitopes were found to have a PI score ≥0.69, indicating a strong possibility for interaction with an antibody due to higher surface accessibility. The linear epitopes predicted by the ABCpred server are also present within the discontinuous segments of B-cell epitopes given by the ElliPro tool. This finding suggests that the predicted B-cell epitopes remain accessible to the surrounding environment even after folding into the TetraFuVac11 3D structure. Although both tools are widely used for B-cell epitope prediction, the ElliPro web server is reported to be more reliable. A comparison study suggested that the accuracy and precision of data obtained through the ElliPro web server are greater than those of the ABCpred tool [73].

Out of the different structural models for interaction between the fusion protein and TLR4 receptors generated by HDOCK, Model 4 was predicted to be the most favourable model for further studies due to a suitable pose and energy profile (pre-simulation) compared to the top 3 models. Although the confidence scores from Model 1 (0.9331), Model 2 (0.9321), and Model 3 (0.9125) were slightly higher than that of Model 4 (0.9071) (S7 Table), their binding energy was predicted to be less favourable by the two different tools. The initial binding free energy of Model 4 was predicted to be −15.9 kcal mol^-1^ by PRODIGY and −15.77 kcal mol^-1^ by HawkDock. In contrast, the binding free energy of the top 3 models was less negative, with Model 2 showing a ΔG value of +16.89 kcal mol^-1^ by HawkDock server. The topological analysis by PDBsum showed that the structural architecture of the TetraFuVac11-TLR4 complex is stabilized by four hydrogen bonds and a dense network of non-bonded hydrophobic interactions. These directional hydrogen bonds are formed between Asp50–Arg51, Glu27–Leu11, Asn35–Ser77, and Glu474–Tyr365. Importantly, the structural stability of the docked complex was maintained by the formation of three salt bridges and an intense network of 239 non-bonded interactions between the vaccine construct and chain A of TLR4. The PRODIGY analysis revealed a network of 11 charged-charged, 16 charged-polar, 30 charged-apolar, 15 polar-polar, 40 polar-apolar, and 25 apolar-apolar interactions. These results are consistent with the previous findings that hydrogen bonds, salt bridges, and interfacial contacts are important to make a stable docked complex [74,75]. These analyses suggest that the docked complex is predominantly stabilized by shape complementarity and a dense network of non-bonded hydrophobic interactions. TetraFuVac11 fusion exclusively interacted with chain A of the TLR4 receptor, as recombinant fusion proteins are too large to interact with both chains simultaneously without clashes. The binding to a single chain of the TLR4 receptor has also been reported previously in other vaccine constructs [76,77].

Molecular dynamics simulations were performed to analyze the structural stability of the TetraFuVac11-TLR4 complex over 100 ns. In the RMSF plot, the first noticeable spike reached ~5.1 Å around the 530 residue position, which corresponds to the C-terminus end of chain A of TLR4. Another dynamic cluster around the 1050–1150 residue position contained a sharp peak reaching up to ~6.5 Å, corresponding to the C-terminus of chain B (Fig 13A). Within multi-chain assemblies, the loop regions and terminal residues tend to have a dynamic conformation, leading to elevated RMSF values [78,79]. As the plot progressed towards the final chain comprising the fusion protein, the RMSF values gradually increased. Peaks seen at the tail of the RMSF plot correspond to the inter-domain flexible linker regions in the fusion protein, as reported previously [80]. The multimeric nature and large size of the docked complex seemed to contribute to the higher RMSD values (Fig 13B). Four chains of TLR4 (chains A-D) and chain E of the fusion protein resulted in a hetero-oligomeric complex with more than 1856 amino acid residues. Large size and multi-chain composition could contribute to elevated RMSD, as reported previously for docked complexes comprising TLR4 and other multi-chain receptors [78,79]. Additionally, a flat SSE timeline also suggests that the domain reorientation and flexible loop dynamics resulted in an elevated global RMSD trend, as the core secondary structural elements remained well conserved over 100 ns of the simulations (Fig 13D). The net binding energy ΔG_bind_, as calculated by MM-GBSA analysis, was predicted to be −248.1 kcal mol^-1^. It was also found that the binding of the docked complex is primarily driven by hydrophobic van der Waals interactions (−375.518 kcal mol ^-1^). The per-frame ΔG_bind_ remained favourable across ten snapshots taken at 10 ns intervals over 100 ns of simulations, fluctuating around a mean of −248.1 kcal mol^-1^.

Normal Mode Analysis (NMA) was performed using iMODS to analyze the stability of the TetraFuVac11-TLR4 vaccine construct during physical movement (Fig 15). In the deformity and B-factor mobility plots, the core binding residues in chain A of the TLR4 (Glu27, Asn35, and Asp50) were located in the stable valleys. An Eigenvalue (1) of 9.367931 × 10^−6^ indicates that the structural stability of the docked complex is strong enough to prevent the dissociation of the vaccine construct from the TLR4 receptor. The elastic network model further validated stable interactions in the form of dark grey, as shown in Fig 15D. The presence of dark red blocks along the central diagonal in the covariance plot suggests that the TetraFuVac11 fusion molecule and TLR4 receptor move in a synchronized manner as a single unit in the docked complex (Fig 15F).

The cytokine profile, as generated by the C-ImmSim web server, showed a quick and high-level release of IFN-ɣ following each of the three injections of the fusion molecule (Fig 16A). The release of IFN-ɣ plays a central role in developing an effective immune response as it leads to the activation of macrophages, promotes antigen presentation, and stimulates other immune cells for microbial killing [81,82]. Similarly, an elevated release of IL-2 after booster doses signifies proliferation of helper T cells (Fig 16A), which is another instrumental component of cell-mediated immunity mechanisms [83]. A low but consistent release of IL-10 corresponds to a favorable immune regulation (Fig 16A), as it prevents excessive activation of T-cells by downregulating antigen presentation [84,85]. The ProtScale analysis predicted 39–44, 53–72, 84–97, 116–124, 134–142, 185–199, 234–260, 336–356, and 380–412 stretches of amino acids to be well surface-exposed, as most of the residues of these stretches showed an accessibility score greater than 6 (Fig 17A). The presence of the majority of Th- cell and B-cell epitopes of TetraFuVac11 in these segments supports their surface accessibility.

The codon-optimized fusion sequence was integrated into the pET-28a(+) vector, as analyzed by SnapGene software, showing a successful generation of the recombinant vector (Fig 18). The probability of soluble expression of the fusion protein, as analyzed by two different tools, SoluProt 1.0 and ProteinSol, showed predicted scores of 0.91 and 0.47, respectively (Fig 19). These values are highly encouraging, as most heterologous proteins tend to yield insoluble expression in *E. coli* [86].

## Conclusion

This study reports an in-depth *in silico* analysis of a novel TetraFuVac11 fusion molecule, as a potential vaccine for tuberculosis. Various epitope prediction tools were used to identify T and B cell epitopes of tnHspX, CFP7, EspC, and tnHrp1. The results for toxicity, antigenicity, allergenicity, and physicochemical attribute predictions were all found to be satisfactory. The predicted 3D structure was refined and validated through the Ramachandran plot, Z-score, and ERRAT score to predict TetraFuVac11 as an energetically favoured and well-folded fusion molecule. Molecular docking and molecular dynamics simulations showed thermodynamically stable interactions between TetraFuVac11 and the TLR4 receptor. Immune simulations predicted an effective immune response to the TetraFuVac11 construct. Surface accessibility analysis for the epitopes in the fusion molecule showed favorable results. Based on the data obtained, the TetraFuVac11 molecule appears to be a promising candidate as a subunit vaccine for tuberculosis. While this study is based on *in silico* analysis, we are currently working on the recombinant production and *in silico* prediction of TetraFuVac11 as a potential subunit vaccine candidate for tuberculosis.

## Supporting information

S1 File Complete amino acid sequence of TetraFuVac11 fusion construct. The linkers are shown in red.
(DOCX)

S2 Fig Comparative analysis of surface accessibility of epitopes in native and truncated HspX and Hrp1. **(A)** Some of the epitopes (shown in black) are buried inside the non-epitopic region in native HspX. (**B)** The epitopes are completely exposed on the surface of the tnHspX. **(C)** In native Hrp1, some of the key epitopes remain deeply buried within the non-epitopic region. (**D)** In the truncated variant, epitopes are freely accessible on the surface to interact with the surrounding environment.
(TIF)

S3 Table List of Th1 cell-specific epitopes of CFP7, HspX and Hrp1 retrieved from the Immune Epitope Database (IEDB) along with their antigenicity, allergenicity and toxicity analyses. 16, 14 and 3 T-cell-specific epitopes were detected for CFP7, HspX and Hrp1, respectively.
(DOCX)

S4 Table List of MHC II binding epitopes of tnHrp1 and EspC, as predicted by the TepiTool web server by IEDB. The tool provided a long list of MHC II binding epitopes, and only epitopes with <1% percentile rank were selected for further *in silico* prediction.
(DOCX)

S5 Table List of Th-cell specific epitopes of EspC and tnHrp1, as predicted by NetMHCIIpan 4.0, along with their antigenicity, allergenicity and toxicity analyses. (DOCX)

S6 Table List of Linear B-cell epitopes of tnHspX, CFP7, EspC, tnHrp1 and fusion protein, as predicted by the ABCpred web server, along with their antigenicity, allergenicity and toxicity analyses. The location of these epitopes in the fusion protein is shown as superscripts.
(DOCX)

S7 Table Top 10 docking models predicted by the HDOCK server, along with the binding free energy values (pre-simulation) as predicted by PRODIGY and HawkDock. (DOCX)
