# Supplementary material for: In silico framework for designing and validating a multi-stage subunit vaccine against Tuberculosis using reverse vaccinology approach
Source: PLoS One. 2026 Aug 27;21(8):e0356788. doi: 10.1371/journal.pone.0356788 (PMC13521415; doi:10.1371/journal.pone.0356788)
Supplement: Supplementary file 1 [file pone.0356788.s001.docx]

LFAAFPSFAGLRPTFDTRLMRLEDEMKEGRYEVRAELPGVDPDKDVDIMVRDGQLTIKAERTEQKDFDGRSEFAYGSFVRTVSLPVGADEDDIKAT**GS**MSQIMYNYPAMLGHAGDMAGYAGTLQSLGAEIAVEQAALQSAWQGDTGITYQAWQAQWNQAMEDLVRAYHAMSSTHEANTMAMMARDTAEAAKWGG**GS**LTAAAQYMREHDIGALPICGDDDRLHGMLTDRDIVIKGLAAGLDPNTATAGELARDSIYYVDANASIQEMLNVMEEHQVRRVPVISEHRLVGIVTEADIARHLPEHAIVQFVKAICSPMA**GS**MTENLTVQPERLGVLASHHDNAAVDASSGVEAAAGLGESVAITHGPYCSQFNDTLNVYLTAHNALGSSLHTAGVDLAKSLRIAAKIYSEADEAWRKAIDGLFT
