# Supplementary material for: In silico framework for designing and validating a multi-stage subunit vaccine against Tuberculosis using reverse vaccinology approach
Source: PLoS One. 2026 Aug 27;21(8):e0356788. doi: 10.1371/journal.pone.0356788 (PMC13521415; doi:10.1371/journal.pone.0356788)
Supplement: Supplementary file 2 [file pone.0356788.s003.docx]

| **Antigen** | **Position** | **Antigenicity (score)** | **Allergenicity** | **Toxicity** |
| --- | --- | --- | --- | --- |
| **CFP7** | | | | |
| LQSLGAEIAVEQAAL | 25-40 | Probable antigen (0.77 ) | Probable non-allergen | Non-toxin |
| AMEDLVRAYHAMSST | 61-75 | Probable non-antigen (0.14 ) | Probable non-allergen | Non-toxin |
| AMMARDTAEAAKWGG | 82-96 | Probable non-antigen (0.04) | Probable allergen | Non-toxin |
| GDMAGYAGTLQSLGA | 16-30 | Probable non-antigen (0.38) | Probable allergen | Non-toxin |
| EDLVRAYHAMSSTHE | 63-77 | Probable non-antigen (0.30) | Probable non-allergen | Non-toxin |
| AQWNQAMEDLVRAYH | 53-70 | Probable non-antigen (0.00) | Probable allergen | Non-toxin |
| DTGITYQAWQAQWNQ | 46-60 | Probable antigen (0.78) | Probable non-allergen | Non-toxin |
| HEANTMAMMARDTAE | 76-90 | Probable non-antigen ( 0.18) | Probable allergen | Non-toxin |
| MAMMARDTAEAAKWG | 81-95 | Probable non-antigen (0.17) | Probable non-allergen | Non-toxin |
| MSQIMYNYPAMLGHA | 1-15 | Probable non-antigen (0.31) | Probable allergen | Non-toxin |
| QAALQSAWQGDTGIT | 36-50 | Probable antigen (0.40) | Probable non-allergen | Non-toxin |
| SAWQGDTGITYQAWQ | 41-55 | Probable non-antigen (0.30) | Probable allergen | Non-toxin |
| VRAYHAMSSTHEANT | 66-80 | Probable antigen (0.031) | Probable non-allergen | Non-toxin |
| YAGTLQSLGAEIAVE | 21-35 | probable antigen (0.80) | Probable allergen | Non-toxin |
| YNYPAMLGHAGDMAG | 6-20 | probable non-antigen (0.36) | Probable non-allergen | Non-toxin |
| YQAWQAQWNQAMEDL | 51-65 | probable antigen (0.49) | Probable allergen | Non-toxin |
| **HspX** | | | | |
| SEFAYGSFVRTVSLPVGADE | 91-110 | Probable non-antigen (0.22) | Probable allergen | Non-toxin |
| AYGSFVRTVSLPVGA | 94-107 | Probable non-antigen (-0.01) | Probable allergen | Non-toxin |
| LFAAFPSFAGLRPTFDTRLM | 21-39 | Probable antigen (0.94) | Probable non-allergen | Non-toxin |
| RTEQKDFDGRSEFAYGSFVR | 81-100 | Probable antigen (0.76 ) | Probable allergen | Non-toxin |
| RDGQLTIKAERTEQKDFDGR | 71-90 | Probable antigen (1.58) | Probable non-allergen | Non-toxin |
| RDGQLTIKAERTEQKDFDGRS | 71-91 | Probable antigen (1.60) | Probable non-allergen | Non-toxin |
| RLEDEMKEGRYEVRAELPGV | 41-60 | Probable antigen (1.20) | Probable allergen | Non-toxin |
| SEFAYGSFVRTVSL | 91-104 | Probable non-antigen (0.12 ) | Probable non-allergen | Non-toxin |
| TVSLPVGADEDDIKAT | 101-116 | Probable antigen (0.98) | Probable non-allergen | Non-toxin |
| GRSEFAYGSFVRTVS | 89-104 | Probable non-antigen (0.18) | Probable allergen | Non-toxin |
| DPDKDVDIMVRDGQLTIKAE | 61-79 | Probable antigen (0.92) | Probable allergen | Non-toxin |
| FAYGSFVRTVSLPVGA | 93-108 | Probable non-antigen (0.04) | Probable allergen | Non-toxin |
| LRPTFDTRLMRLEDEMKEGRYE | 31-52 | Probable antigen (1.34) | Probable non-allergen | Non-toxin |
| YEVRAELPGVDPDKDVDIMV | 51-70 | Probable antigen  (1.11 ) | Probable allergen | Non-toxin |
| **tnHrp1** | | | | |
| YMREHDIGA | 27-35 | Probable antigen (1.05). | Probable allergen | Non-toxin |
| GLDPNTATA | 62-70 | Probable antigen (1.7) | Probable non-allergen | Non-toxin |
| IYYVDANAS | 78-85 | Probable antigen (1.0) | Probable non-allergen | Non-toxin |
