# Supplementary material for: In silico framework for designing and validating a multi-stage subunit vaccine against Tuberculosis using reverse vaccinology approach
Source: PLoS One. 2026 Aug 27;21(8):e0356788. doi: 10.1371/journal.pone.0356788 (PMC13521415; doi:10.1371/journal.pone.0356788)
Supplement: Supplementary file 3 [file pone.0356788.s004.docx]

| **tnHrp1** | | | |
| --- | --- | --- | --- |
| **Allele** | **Location** | **Peptide** | **Score/percentile rank** |
| HLA-DQA1*03:01/DQB1*03:02 | 55-69 | RDSIYYVDANASIQE | 0.0449/0.12 |
| HLA-DRB1*04:01 |  |  | 0.9294/0.14 |
| HLA-DQA1*03:01/DQB1*03:02 | 87-101 | EHRLVGIVTEADIAR | 0.0401/0.15 |
| HLA-DRB1*04:05 |  |  | 0.9354/0.16 |
| HLA-DRB1*04:05 | 86-100 | SEHRLVGIVTEADIA | 0.9132/0.24 |
| HLA-DQA1*03:01/DQB1*03:02 |  |  | 0.0355/0.28 |
| HLA-DRB1*04:01 | 54-68 | ARDSIYYVDANASIQ | 0.8683/0.29 |
| HLA-DQA1*03:01/DQB1*03:02 |  |  | 0.0339/0.33 |
| HLA-DRB1*04:01 | 56-70 | DSIYYVDANASIQEM | 0.864/0.30 |
| HLA-DRB1*04:01 | 3-17 | AAAQYMREHDIGALP | 0.8504/0.37 |
| HLA-DRB1*04:01 | 4-18 | AAQYMREHDIGALPI | 0.8396/0.44 |
| HLA-DQA1*03:01/DQB1*03:02 | 85-99 | ISEHRLVGIVTEADI | 0.0296/0.47 |
| HLA-DRB1*04:05 |  |  | 0.8432/0.57 |
| HLA-DRB1*11:01 | 81-95 | RVPVISEHRLVGIVT | 0.8747/0.58 |
| HLA-DRB1*11:01 | 80-94 | RRVPVISEHRLVGIV | 0.8436/0.69 |
| HLA-DQA1*05:01/DQB1*02:01 | 55-69 | RDSIYYVDANASIQE  RDSIYYVDANASIQE | 0.5714/0.71 |
| HLA-DRB1*04:05 |  |  | 0.8095/0.76 |
| HLA-DQA1*01:02/DQB1*06:02 | 43-57 | LDPNTATAGELARDS | 0.614/0.72 |
| HLA-DRB1*04:05 | 88-102 | HRLVGIVTEADIARH | 0.7938/0.81 |
| HLA-DQA1*05:01/DQB1*02:01 | 42-56 | GLDPNTATAGELARD | 0.5399/0.84 |
| HLA-DQA1*03:01/DQB1*03:02 | 56-70 | DSIYYVDANASIQEM | 0.0234/0.86 |
| HLA-DRB1*01:01 | 55-69 | RDSIYYVDANASIQE | 0.8106/0.87 |
| HLA-DQA1*03:01/DQB1*03:02 | 53-67 | LARDSIYYVDANASI | 0.0225/0.93 |
| EspC | | | |
| HLA-DQA1*05:01/DQB1*02:01 | 82-96 | IAAKIYSEADEAWRK | 0.8375/0.06 |
| HLA-DQA1*05:01/DQB1*02:01 | 83-97 | AAKIYSEADEAWRKA | 0.8419/0.06 |
| HLA-DQA1*01:02/DQB1*06:02 | 61-75 | AHNALGSSLHTAGVD | 0.8113/0.08 |
| HLA-DRB1*11:01 | 77-91 | AKSLRIAAKIYSEAD | 0.9586/0.08 |
| HLA-DQA1*05:01/DQB1*02:01 | 81-95 | RIAAKIYSEADEAWR | 0.7661/0.12 |
| HLA-DRB1*07:01 | 71-85 | TAGVDLAKSLRIAAK | 0.896/0.17 |
| HLA-DQA1*05:01/DQB1*02:01 | 84-98 | AKIYSEADEAWRKAI | 0.7162/0.23 |
| HLA-DQA1*01:02/DQB1*06:02 | 18-32 | HHDNAAVDASSGVEA | 0.7358/0.23 |
| HLA-DQA1*03:01/DQB1*03:02 | 83-97 | AAKIYSEADEAWRKA | 0.0366/0.24 |
| HLA-DRB1*09:01 | 71-85 | TAGVDLAKSLRIAAK | 0.8155/0.24 |
| HLA-DRB1*11:01 | 76-90 | LAKSLRIAAKIYSEA | 0.9312/0.24 |
| HLA-DRB1*13:01 | 77-91 | AKSLRIAAKIYSEAD | 0.884/0.25 |
| HLA-DQA1*03:01/DQB1*03:02 | 82-96 | IAAKIYSEADEAWRK | 0.0361/0.27 |
| HLA-DRB1*07:01 | 70-84 | HTAGVDLAKSLRIAA | 0.8582/0.28 |
| HLA-DRB1*04:01 | 54-68 | TLNVYLTAHNALGSS | 0.8686/0.29 |
| HLA-DQA1*01:02/DQB1*06:02 | 24-38 | VDASSGVEAAAGLGE | 0.7148/0.3 |
| HLA-DQA1*01:02/DQB1*06:02 | 60-74 | TAHNALGSSLHTAGV | 0.6948/0.35 |
| HLA-DQA1*01:02/DQB1*06:02 | 17-31 | SHHDNAAVDASSGVE | 0.6968/0.35 |
| HLA-DRB1*09:01 | 70-84 | HTAGVDLAKSLRIAA | 0.7627/0.44 |
| HLA-DQA1*03:01/DQB1*03:02 | 81-95 | RIAAKIYSEADEAWR | 0.028/0.54 |
| HLA-DRB1*13:01 | 76-90 | LAKSLRIAAKIYSEA | 0.8208/0.55 |
| HLA-DRB1*07:01 | 69-83 | LHTAGVDLAKSLRIA | 0.7823/0.57 |
| HLA-DRB1*04:01 | 55-69 | LNVYLTAHNALGSSL | 0.8141/0.58 |
| HLA-DRB1*11:01 | 78-92 | KSLRIAAKIYSEADE | 0.8613/0.6 |
| HLA-DQA1*01:02/DQB1*06:02 | 62-76 | HNALGSSLHTAGVDL | 0.6305/0.64 |
| HLA-DQA1*01:02/DQB1*06:02 | 67-81 | SSLHTAGVDLAKSLR | 0.622/0.69 |
| HLA-DQA1*01:02/DQB1*06:02 | 68-82 | SLHTAGVDLAKSLRI | 0.6169/0.7 |
| HLA-DQA1*05:01/DQB1*02:01 | 80-94 | LRIAAKIYSEADEAW | 0.5692/0.72 |
| HLA-DRB1*11:01 | 75-89 | DLAKSLRIAAKIYSE | 0.8381/0.74 |
| HLA-DQA1*01:02/DQB1*06:02 | 23-37 | AVDASSGVEAAAGLG | 0.6046/0.78 |
| HLA-DQA1*03:01/DQB1*03:02 | 65-79 | LGSSLHTAGVDLAKS | 0.0238/0.81 |
| HLA-DQA1*01:02/DQB1*06:02 | 19-33 | HDNAAVDASSGVEAA | 0.5867/0.83 |
| HLA-DRB1*15:01 | 71-85 | TAGVDLAKSLRIAAK | 0.7125/0.88 |
| HLA-DQA1*01:02/DQB1*06:02 | 59-73 | LTAHNALGSSLHTAG | 0.5686/0.96 |
