# Supplementary material for: In silico framework for designing and validating a multi-stage subunit vaccine against Tuberculosis using reverse vaccinology approach
Source: PLoS One. 2026 Aug 27;21(8):e0356788. doi: 10.1371/journal.pone.0356788 (PMC13521415; doi:10.1371/journal.pone.0356788)
Supplement: Supplementary file 4 [file pone.0356788.s005.docx]

| **Epitope** | **Alleles** | **Score/rank** | **Position** | **Antigenicity** | **Allergenicity** | **Toxicity** |
| --- | --- | --- | --- | --- | --- | --- |
| **EspC** | | | | | | |
| TAGVDLAKSLRIAAK | DRB1_0901 | 0.75/0.30 | 71-85 | Probable antigen  (0.67) | Probable non-allergen | Non-toxin |
|  | DRB1_0701 | 0.87/0.22 |  |  |  |  |
| AKSLRIAAKIYSEAD | DRB1_1101 | 0.96/0.13 | 77-91 | Probable antigen (0.70) | Probable non-allergen | Non-toxin |
|  | DRB1_1301 | 0.86/0.26 |  |  |  |  |
| LAKSLRIAAKIYSEA | DRB1_1101 | 0.94/0.23 | 76-90 | Probable non-antigen (0.33) | Probable non-allergen | Non-toxin |
|  | DRB1_1301 | 0.80/0.56 |  |  |  |  |
| HTAGVDLAKSLRIAA | DRB1_0901 | 0.68/0.69 | 70-84 | Probable antigen (0.81) | Probable non-allergen | Non-toxin |
|  | DRB1_0701 | 0.82/0.34 |  |  |  |  |
| TLNVYLTAHNALGSS | DRB1_0401 | 0.89/0.16 | 54-68 | Probable antigen (1.16) | Probable non-allergen | Non-toxin |
| LHTAGVDLAKSLRIA | DRB1_0701 | 0.72/0.61 | 70-84 | Probable antigen (0.75) | Probable non-allergen | Non-toxin |
| LNVYLTAHNALGSSL | DRB1_0401 | 0.85/0.29 | 55-69 | Probable antigen (1.07) | Probable allergen | Non-toxin |
| DLAKSLRIAAKIYSE | DRB1_1101 | 0.88/0.51 | 75-89 | Probable non-antigen (0.21) | Probable non-allergen | Non-toxin |
| **tnHrp1** | | | | | | |
| RDSIYYVDANASIQE | DRB1_0401 | 0.94/0.07 | 55-69 | Probable non-antigen (0.25) | Probable allergen | Non-toxin |
|  | DRB1_0405 | 0.74/0.68 |  |  |  |  |
|  | DRB1_0701 | 0.64/0.94 |  |  |  |  |
|  | DRB1_0901 | 0.72/0.54 |  |  |  |  |
| SEHRLVGIVTEADIA | DRB1_0405 | 0.89/0.20 | 86-100 | Probable Non-antigen (0.10) | Probable non-allergen | Non-toxin |
| ARDSIYYVDANASIQ | DRB1_0901 | 0.63/0.97 | 54-68 | Probable non-antigen (0.31) | Probable non-allergen | Non-toxin |
|  | DRB1_0401 | 0.88/0.19 |  |  |  |  |
| AAAQYMREHDIGALP | DRB1_0401 | 0.86/0.24 | 3-17 | Probable non-antigen (0.38) | Probable non-allergen | Non-toxin |
| AAQYMREHDIGALPI | DRB1_0401 | 0.85/0.28 | 4-18 | Probable antigen (0.54) | Probable allergen | Non-toxin |
| ISEHRLVGIVTEADI | DRB1_0405 | 0.80/0.46 | 85-99 | Probable non-antigen (0.05) | Probable non-allergen | Non-toxin |
| RVPVISEHRLVGIVT | DRB1_1101 | 0.82/0.79 | 81-95 | Probable non-antigen (-0.42) | Probable allergen | Non-toxin |
| RRVPVISEHRLVGIV | DRB1_1101 | 0.80/0.91 | 80-94 | Probable non-antigen  (-0.36) | Probable allergen | Non-toxin |
| DSIYYVDANASIQEM | DRB1_0401 | 0.89/0.17 | 56-70 | Probable non-antigen (0.33) | Probable non-allergen | Non-toxin |
| GLDPNTATAGELARD | DQB1_0201 | 0.53/0.84 | 42-56 | Probable antigen (0.98) | Probable non-allergen | Non-toxin |
