# Supplementary material for: In silico framework for designing and validating a multi-stage subunit vaccine against Tuberculosis using reverse vaccinology approach
Source: PLoS One. 2026 Aug 27;21(8):e0356788. doi: 10.1371/journal.pone.0356788 (PMC13521415; doi:10.1371/journal.pone.0356788)
Supplement: Supplementary file 5 [file pone.0356788.s006.docx]

| **Epitope Sequence** | **Score** | **Antigenicity** | **Allergenicity** | **Toxicity** |
| --- | --- | --- | --- | --- |
| **tnHspX** | | | | |
| D^24^EMKEGRYEVRAELPG^39^ | 0.94 | Probable antigen (1.10) | Probable allergen | Non-toxin |
| V^33^RAELPGVDPDKDVDI^48^ | 0.88 | Probable antigen (1.12) | Probable non-allergen | Non-toxin |
| P^6^SFAGLRPTFDTRLMR^21^ | 0.84 | Probable antigen (1.17) | Probable non-allergen | Non-toxin |
| I^48^MVRDGQLTIKAERTE^63^ | 0.83 | Probable antigen (94) | Probable non-allergen | Non-toxin |
| A^9^GLRPTFDTRLMRLED^24^ | 0.80 | Probable antigen (1.37) | Probable non-allergen | Non-toxin |
| M^20^RLEDEMKEGRYEVRA^35^ | 0.79 | Probable antigen (1.48) | Probable non-allergen | Non-toxin |
| T^17^RLMRLEDEMKGRYE^32^ | 0.78 | Probable antigen ( 0.96) | Probable allergen | Non-toxin |
| L^11^RPTFDTRLMRLEDEM^26^ | 0.78 | Probable antigen (1.34) | Probable allergen | Non-toxin |
| L^22^EDEMKEGRYEVRAEL^37^ | 0.76 | Probable antigen  (1.19) | Probable allergen | Non-toxin |
| Q^54^LTIKAERTEQKDFDG^69^ | 0.74 | Probable antigen (1.45) | Probable non-allergen | Non-toxin |
| G^39^VDPDKDVDIMVRDGQ^54^ | 0.74 | Probable antigen (0.96) | Probable non-allergen | Non-toxin |
| Y^31^EVRAELPGVDPDKDV^46^ | 0.74 | Probable antigen ( 1.03) | Probable non-allergen | Non-toxin |
| V^79^RTVSLPVGADEDDIK^94^ | 0.73 | Probable antigen (0.72) | Probable non-allergen | Non-toxin |
| K^27^EGRYEVRAELPGVDP^42^ | 0.70 | Probable antigen (1.23) | Probable non-allergen | Non-toxin |
| **CFP7** | | | | |
| A^140^WQGDTGITYQAWQAQ^155^ | 0.93 | Probable antigen (0.52) | Probable allergen | Non-toxin |
| G^143^DTGITYQAWQAQWNQ^158^ | 0.91 | Probable antigen (0.95) | Probable allergen | Non-toxin |
| P^107^AMLGHAGDMAGYAGT^123^ | 0.85 | Probable antigen (0.40) | Probable non-allergen | Non-toxin |
| I^130^AVEQAALQSAWQGDT^145^ | 0.83 | Probable antigen (0.42) | Probable non-allergen | Non-toxin |
| L^163^VRAYHAMSSTHEANT^177^ | 0.81 | Probable antigen (0.74). | Probable non-allergen | Non-toxin |
| L^137^QSAWQGDTGITYQAW^152^ | 0.80 | Probable non-antigen (0.39) | Probable allergen | Non-toxin |
| Q^138^SAWQGDTGITYQAWQ^153^ | 0.78 | Probable non-antigen (0.36) | Probable allergen | Non-toxin |
| D^115^MAGYAGTLQSLGAEI^130^ | 0.77 | Probable antigen (0.70) | Probable non-allergen | Non-toxin |
| M^99^SQIMYNYPAMLGHAG^114^ | 0.77 | Probable non-antigen (0.24) | Probable non-allergen | Non-toxin |
| Q^158^AMEDLVRAYHAMSST^173^ | 0.75 | Probable non-antigen (0.14) | Probable non-allergen | Non-toxin |
| G^146^ITYQAWQAQWNQAME^161^ | 0.75 | Probable antigen (0.81) | Probable allergen | Non-toxin |
| S^172^THEANTMAMMARDTA^187^ | 0.73 | Probable non-antigen (0.33) | Probable non-allergen | Non-toxin |
| S^100^QIMYNYPAMLGHAGD^115^ | 0.73 | Probable non-antigen (0.35) | Probable non-allergen | Non-toxin |
| **tnHrp1** | | | | |
| T^198^AAAQY140MREHDIGALP^213^ | 0.94 | Probable antigen (0.42) | Probable non-allergen | Non-toxin |
| P^213^ICGDDDRLHGMLTDR^228^ | 0.90 | Probable antigen (0.71). | Probable allergen | Non-toxin |
| H^222^GMLTDRDIVIKGLAA^236^ | 0.86 | Probable non-antigen (0.30) | Probable allergen | Non-toxin |
| N^268^VMEEHQVRRVPVISE^283^ | 0.82 | Probable non-antigen (0.28) | Probable non-allergen | Non-toxin |
| A^211^LPICGDDDRLHGMLT^226^ | 0.82 | Probable antigen (0.54). | Probable allergen | Non-toxin |
| E^248^LARDSIYYVDANASI^263^ | 0.81 | Probable non-antigen (0.06) | Probable non-allergen | Non-toxin |
| A^250^RDSIYYVDANASIQE^265^ | 0.80 | Probable non-antigen (0.28) | Probable allergen | Non-toxin |
| M^204^REHDIGALPICGDDD^219^ | 0.76 | Probable non-antigen (0.34) | Probable allergen | Non-toxin |
| G^247^ELARDSIYYVDANAS^262^ | 0.76 | Probable non-antigen (0.04) | Probable non-allergen | Non-toxin |
| K^233^GLAAGLDPNTATAGE^248^ | 0.76 | Probable antigen (1.23) | Probable non-allergen | Non-toxin |
| L^212^PICGDDDRLHGMLTD^227^ | 0.74 | Probable antigen (0.62) | Probable allergen | Non-toxin |
| E^292^ADIARHLPEHAIVQF^307^ | 0.73 | Probable non-antigen (0.18) | Probable allergen | Non-toxin |
| P^279^VISEHRLVGIVTEAD^294^ | 0.73 | Probable non-antigen (-0.00) | Probable allergen | Non-toxin |
| Q^202^YMREHDIGALPICGD^217^ | 0.73 | Probable non-antigen (0.25) | Probable allergen | Non-toxin |
| Y^256^VDANASIQEMLNVME^271^ | 0.71 | Probable non-antigen (0.34) | Probable non-allergen | Non-toxin |
| A^246^GELARDSIYYVDANA^261^ | 0.71 | Probable non-antigen (0.05) | Probable non-allergen | Non-toxin |
| H^284^RLVGIVTEADIARHL^299^ | 0.70 | Probable non-antigen (-0.27) | Probable non-allergen | Non-toxin |
| **EspC** | | | | |
| A^241^VDASSGVEAAAGLGE^256^ | 0.89 | Probable antigen (1.18) | Probable allergen | Non-toxin |
| E^328^RLGVLASHDNAAVD^343^ | 0.86 | Probable non-antigen (0.10) | Probable allergen | Non-toxin |
| S^357^VAITHGPYCSQFNDT^372^ | 0.84 | Probable antigen (0.45) | Probable non-allergen | Non-toxin |
| P^364^YCSQFNDTLNVYLTA^379^ | 0.77 | Probable antigen (0.66) | Probable non-allergen | Non-toxin |
| I^360^THGPYCSQFNDTLNV^375^ | 0.77 | Probable antigen (0.50) | Probable allergen | Non-toxin |
| E^356^SVAITHGPYCSQFND^371^ | 0.76 | Probable non-antigen (0.28) | Probable non-allergen | Non-toxin |
| N^374^VYLTAHNALGSSLHT^389^ | 0.75 | Probable antigen (1.05) | Probable non-allergen | Non-toxin |
| A^402^KIYSEADEAWRKAID^417^ | 0.74 | Probable antigen (0.41) | Probable allergen | Non-toxin |
| A^401^AKIYSEADEAWRKAI^416^ | 0.74 | Probable non-antigen (0.13) | Probable allergen | Non-toxin |
| G^353^LGESVAITHGPYCSQ^368^ | 0.74 | Probable antigen (0.64) | Probable non-allergen | Non-toxin |
| G^331^VLASHDNAAVDASS^346^ | 0.70 | Probable antigen (0.46) | Probable non-allergen | Non-toxin |
| A^379^HNALGSSLHTAGVDL^394^ | 0.70 | Probable antigen (1.38) | Probable allergen | Non-toxin |
| **TetraFuVac11** | | | | |
| D²⁴EMKEGRYEVRAELPG³⁹ | 0.94 | Probable antigen (1.10) | Probable allergen | Non-toxin |
| T^198^AAAQYMREHDIGALP^213^ | 0.94 | Probable antigen (0.42) | Probable non-allergen | Non-toxin |
| A¹⁴⁰WQGDTGITYQAWQAQ¹⁵⁵ | 0.93 | Probable antigen (0.52) | Probable allergen | Non-toxin |
| G¹⁴³DTGITYQAWQAWNQ¹⁵⁷ | 0.91 | Probable non-antigen (0.30) | Probable allergen | Non-toxin |
| P²¹³ICGDDDRLHGMLTDR²²⁸ | 0.90 | Probable antigen (0.71) | Probable allergen | Non-toxin |
| R¹⁸⁴DTAEAAKWGGGSLTA¹⁹⁹ | 0.90 | Probable antigen (0.68) | Probable non-allergen | Non-toxin |
| A³⁴¹VDASSGVEAAAGLGE³⁵⁶ | 0.89 | Probable antigen (1.18) | Probable allergen | Non-toxin |
| V³³RAELPGVDPDKDVDI⁴⁸ | 0.88 | Probable antigen (1.12) | Probable non-allergen | Non-toxin |
| A³¹⁰ICSPMAGSMTENLTV³²⁵ | 0.87 | Probable antigen (0.56) | Probable allergen | Non-toxin |
| E³²⁸RLGVLASHHDNAAVD³⁴³ | 0.86 | Probable non-antigen (0.13) | Probable allergen | Non-toxin |
| H²²²GMLTDRDIVIKGLAA²³⁷ | 0.86 | Probable non-antigen (0.30) | Probable allergen | Non-toxin |
| P¹⁰⁷AMLGHAGDMAGYAGT¹²² | 0.85 | Probable antigen (0.40) | Probable non-allergen | Non-toxin |
| P⁶SFAGLRPTFDTRLMR²¹ | 0.84 | Probable antigen (1.17) | Probable non-allergen | Non-toxin |
| L¹⁶³VRAYHAMSSTHEANT¹⁷⁸ | 0.81 | Probable antigen (0.74) | Probable non-allergen | Non-toxin |
| S³⁵⁷VAITHGPYCSQFNDT³⁷² | 0.84 | Probable antigen (0.45) | Probable non-allergen | Non-toxin |
| I⁴⁸MVRDGQLTIKAERTE⁶³ | 0.83 | Probable antigen (0.94) | Probable non-allergen | Non-toxin |
| I¹³⁰AVEQAALQSAWQGDT¹⁴⁵ | 0.83 | Probable antigen (0.42) | Probable non-allergen | Non-toxin |
| N²⁶⁸VMEEHQVRRVPVISE²⁸³ | 0.82 | Probable non-antigen (0.28) | Probable non-allergen | Non-toxin |
| A²¹¹LPICGDDDRLHGMLT²²⁶ | 0.82 | Probable antigen (0.54) | Probable allergen | Non-toxin |
| E²⁴⁸LARDSIYYVDANASI²⁶³ | 0.81 | Probable non-antigen (0.06) | Probable non-allergen | Non-toxin |
| G¹⁹⁴GSLTAAAQYMREHDI²⁰⁹ | 0.81 | Probable antigen (0.73) | Probable allergen | Non-toxin |
| A⁹GLRPTFDTRLMRLED²⁴ | 0.80 | Probable antigen (1.37) | Probable non-allergen | Non-toxin |
| V⁸²SLPVGADEDDIKATG⁹⁷ | 0.80 | Probable antigen (0.88) | Probable non-allergen | Non-toxin |
| A²⁵⁰RDSIYYVDANASIQE²⁶⁵ | 0.80 | Probable non-antigen (0.28) | Probable allergen | Non-toxin |
| L¹³⁷QSAWQGDTGITYQAW¹⁵² | 0.80 | Probable non-antigen (0.39) | Probable allergen | Non-toxin |
| E⁹⁰DDIKATGSMSQIMYN¹⁰⁵ | 0.79 | Probable antigen (0.81) | Probable allergen | Non-toxin |
| M²⁰RLEDEMKEGRYEVRA³⁵ | 0.79 | Probable antigen (1.48) | Probable non-allergen | Non-toxin |
| T¹⁷RLMRLEDEMKEGRYE³² | 0.78 | Probable antigen (0.89) | Probable non-allergen | Non-toxin |
| Q¹³⁸SAWQGDTGITYQAWQ¹⁵³ | 0.78 | Probable non-antigen (0.36) | Probable allergen | Non-toxin |
| L¹¹RPTFDTRLMRLEDEM²⁶ | 0.78 | Probable antigen (1.34) | Probable allergen | Non-toxin |
| M⁹⁹SQIMNYVPAMLGHAG¹¹⁴ | 0.77 | Probable non-antigen (0.11) | Probable allergen | Non-toxin |
| P³⁶⁴YCSQFNDILNVYLTA³⁷⁹ | 0.77 | Probable antigen (0.40) | Probable allergen | Non-toxin |
| I³⁶⁰THGPYCSQFNDILNV³⁷⁵ | 0.77 | Probable non-antigen (0.14) | Probable non-allergen | Non-toxin |
| M¹⁸¹MARDTAEAAKWGGGS¹⁹⁶ | 0.77 | Probable antigen (0.65) | Probable non-allergen | Non-toxin |
| D¹¹⁵MAGYAGTQSLGAEI¹²⁹ | 0.77 | Probable antigen (1.04) | Probable non-allergen | Non-toxin |
| E³⁵⁶SVAITHGPYCSQFND³⁷¹ | 0.76 | Probable non-antigen (0.28) | Probable non-allergen | Non-toxin |
| V³⁰⁸KAICSPMAGSMTENL³²³ | 0.76 | Probable non-antigen (0.37) | Probable allergen | Non-toxin |
| G²⁴⁷ELARDSIYYVDANAS²⁶² | 0.76 | Probable non-antigen (0.04) | Probable non-allergen | Non-toxin |
| K²³³GLAAGLDPNTATAGE²⁴⁸ | 0.76 | Probable antigen (1.23) | Probable non-allergen | Non-toxin |
| L²²EDEMKEGRYEVRAEL³⁷ | 0.76 | Probable antigen (1.19) | Probable allergen | Non-toxin |
| M²⁰⁴REHDIGALPICGDDD²¹⁹ | 0.76 | Probable non-antigen (0.34) | Probable allergen | Non-toxin |
| N³⁷⁴VYLTAHNALGSSLHT³⁸⁹ | 0.75 | Probable antigen (1.05) | Probable non-allergen | Non-toxin |
| Q¹⁵⁸AMEDLVRAYHAMSST¹⁷³ | 0.75 | Probable non-antigen (0.14) | Probable non-allergen | Non-toxin |
| G¹⁴⁶ITYQAWQAWNQAME¹⁶⁰ | 0.75 | Probable non-antigen (0.15) | Probable non-allergen | Non-toxin |
| Q⁵⁴LTIKAERTEQKDFDG⁶⁹ | 0.74 | Probable antigen (1.45) | Probable non-allergen | Non-toxin |
| A⁴⁰¹KIYSEADEAWRKAID⁴¹⁶ | 0.74 | Probable antigen (0.41) | Probable allergen | Non-toxin |
| G³⁹VDPDKDVDIMVRDGQ⁵⁴ | 0.74 | Probable antigen (0.96) | Probable non-allergen | Non-toxin |
| G³⁵³LGESVAITHGPYCSQ³⁶⁸ | 0.74 | Probable antigen (0.64) | Probable non-allergen | Non-toxin |
| C³¹²SPMAGSMTENLTVQP³²⁷ | 0.74 | Probable antigen (0.82) | Probable allergen | Non-toxin |
| Y³¹EVRAELPGVDPDKDV⁴⁶ | 0.74 | Probable antigen (1.03) | Probable non-allergen | Non-toxin |
| V³⁰⁵QFVKAICSPMAGSMT³²⁰ | 0.74 | Probable non-antigen (0.35) | Probable non-allergen | Non-toxin |
| L²¹²PICGDDDRLHGMLTD²²⁷ | 0.74 | Probable antigen (0.62) | Probable allergen | Non-toxin |
| V⁷⁹RTVSLPVGADEDDIK⁹⁴ | 0.73 | Probable antigen (0.72) | Probable non-allergen | Non-toxin |
| E²⁹²ADIARHLPEHAIVQF³⁰⁷ | 0.73 | Probable non-antigen (0.18) | Probable allergen | Non-toxin |
| P²⁷⁹VISEHRLVGIVTEAD²⁹⁴ | 0.73 | Probable non-antigen (0.00) | Probable allergen | Non-toxin |
| Q²⁰²YMREHDIGALPICGD²¹⁷ | 0.73 | Probable non-antigen (0.25) | Probable allergen | Non-toxin |
| S¹⁷²THEANTMAMMARDTA¹⁸⁷ | 0.73 | Probable non-antigen (0.33) | Probable non-allergen | Non-toxin |
| S¹⁰⁰QIMNYVPAMLGHAGD¹¹⁵ | 0.73 | Probable non-antigen (0.26) | Probable allergen | Non-toxin |
| G³³¹VLASHHDNAAVDASS³⁴⁶ | 0.71 | Probable antigen (0.46) | Probable non-allergen | Non-toxin |
| Y²⁵⁶VDANASIQEMLNVME²⁷¹ | 0.71 | Probable non-antigen (0.34) | Probable non-allergen | Non-toxin |
| A²⁴⁶GELARDSIYYVDANA²⁶¹ | 0.71 | Probable non-antigen (0.05) | Probable non-allergen | Non-toxin |
| G⁸⁷ADEDDIKATGSMSQI¹⁰² | 0.70 | Probable antigen (1.14) | Probable non-allergen | Non-toxin |
| A³⁷⁹HNALGSSLLTAGVDL³⁹⁴ | 0.70 | Probable antigen (0.86) | Probable allergen | Non-toxin |
| H²⁸⁴RLVGIVTEADIARHL²⁹⁹ | 0.70 | Probable non-antigen (-0.27) | Probable non-allergen | Non-toxin |
| K²⁷EGRYEVRAELPGVDP⁴² | 0.70 | Probable antigen (1.23) | Probable non-allergen | Non-toxin |
| A¹⁸⁷EAAKWGGSLTAAAQ²⁰¹ | 0.70 | Probable non-antigen (0.30) | Probable allergen | Non-toxin |
