# Supplementary material for: In silico framework for designing and validating a multi-stage subunit vaccine against Tuberculosis using reverse vaccinology approach
Source: PLoS One. 2026 Aug 27;21(8):e0356788. doi: 10.1371/journal.pone.0356788 (PMC13521415; doi:10.1371/journal.pone.0356788)
Supplement: Supplementary file 6 [file pone.0356788.s007.docx]

| Rank | Docking Score | Confidence Score | Binding Free Energy (Kcal mol^-1^) | |
| --- | --- | --- | --- | --- |
|  | | | HawkDock | PRODIGY |
| 1 | -281.78 | 0.9331 | -4.86 | -14.3 |
| 2 | -281.00 | 0.9321 | +16.89 | -13.1 |
| 3 | -267.20 | 0.9125 | -11.0 | -12.9 |
| 4 | -263.91 | 0.9071 | -15.77 | -15.9 |
| 5 | -257.65 | 0.8959 | +6 | -10.03 |
| 6 | -257.49 | 0.8957 | -4.98 | -15.5 |
| 7 | -254.34 | 0.8896 | -56.94 | -14.6 |
| 8 | -254.07 | 0.8891 | -25.47 | -13.6 |
| 9 | -253.95 | 0.8888 | -30.96 | -14.0 |
| 10 | -251.92 | 0.8848 | +1.27 | -12.9 |
